# Bats distress vocalizations carry fast amplitude modulations that could represent an acoustic correlate of roughness

**DOI:** 10.1038/s41598-020-64323-7

**Published:** 2020-04-30

**Authors:** Julio C. Hechavarría, M. Jerome Beetz, Francisco García-Rosales, Manfred Kössl

**Affiliations:** 1 Institut für Zellbiologie und Neurowissenschaft, Goethe-Universität, Frankfurt/M., Germany; 2 Zoology II Emmy-Noether Animal Navigation Group, Biocenter, University of Würzburg, Würzburg, Germany

**Keywords:** Neurophysiology, Animal behaviour, Animal physiology

## Abstract

Communication sounds are ubiquitous in the animal kingdom, where they play a role in advertising physiological states and/or socio-contextual scenarios. Human screams, for example, are typically uttered in fearful contexts and they have a distinctive feature termed as “roughness”, which depicts amplitude fluctuations at rates from 30–150 Hz. In this article, we report that the occurrence of fast acoustic periodicities in harsh sounding vocalizations is not unique to humans. A roughness-like structure is also present in vocalizations emitted by bats (species *Carollia perspicillata*) in distressful contexts. We report that 47.7% of distress calls produced by bats carry amplitude fluctuations at rates ~1.7 kHz (>10 times faster than temporal modulations found in human screams). In bats, rough-like vocalizations entrain brain potentials and are more effective in accelerating the bats’ heart rate than slow amplitude modulated sounds. Our results are consistent with a putative role of fast amplitude modulations (roughness in humans) for grabbing the listeners attention in situations in which the emitter is in distressful, potentially dangerous, contexts.

## Introduction

The ability to communicate fear and discomfort using sounds is ubiquitous in vertebrates. Humans, for example, produce fearful screams to advertise the presence of uncomfortable socio-contextual scenarios, such as dangerous situations that could lead to potential harm. In humans, recognizing sounds as fearful is linked to an acoustic feature defined as “roughness”, i.e. amplitude modulations (AMs) in the sounds uttered occurring at frequencies between 30–150 Hz^1,2^. In addition to naturalistic screaming, in humans, roughness is also found in infant cries^3^, in harsh sounds produced in musical compositions such as the opera and hard rock^4,5^, as well as in sounds used in artificial alarm systems^1^.

The ability to produce screams is not unique to humans^6–11^, but, at present, we do not know whether human and non-human animals rely on similar strategies to create harsh sounding, alarm vocalizations. The word “rough” has been used in several studies to describe agonistic vocalizations of animal groups including non-human primates, otters, and birds, among others^12–17^. In those species, the acoustic correlates of roughness were not studied quantitatively using methods similar to those employed for characterizing the human soundscape. Thus, it is unclear whether the acoustic correlates of roughness observed in non-human animals are comparable to those described in studies on humans. Answering this question is important, as it would allow us to assess whether roughness is an evolutionarily preserved acoustic regime of mammalian vocalizations produced in fearful contexts or a unique feature of human vocalizations. In other words, roughness could be a shared feature of animal alarm and fearful vocalizations.

To test this idea, in this article, we searched for fast amplitude fluctuations -the acoustic correlate of roughness - in distress vocalizations emitted by bats (species: *Carollia perspicillata*). Note that throughout this article, we refer to fast amplitude modulated or rough-like sounds and not to “rough” sounds in the same sense they have been described in humans^1^. The reason for this distinction is simple: we can only speculate about what non-human animals perceive when listening to fast amplitude modulated vocalizations.

Bats constitute a highly vocal animal group that relies on sounds for navigation (echolocation) and inter-individual communication^18–20^. Bats emit distress calls when tangled in catch nets or caught by a predator or a person^21–24^. Bat distress vocalizations are typically noisy and broadband and trigger exploratory and mobbing behaviors in individuals from the same and other species^22,23,25,26^. Bats utter distress calls in “sequences” composed of many syllables^24,27–30^, making these sounds ideal for exploring whether rough-like sounds occur at preferred sequence positions. Bat distress vocalizations are known to evoke strong neural responses in the amygdala^31^, to entrain field potentials and spiking in the auditory cortex^27,28,30^, and to boost activity in the hypothalamic-pituitary axes^32,33^.

Bat distress vocalizations share functional similarities with fearful human screams, in the sense that both have the potential of influencing the behavior and physiology of listeners while the broadcaster faces a distressful, potentially dangerous, context. Following this idea, and assuming that fast amplitude modulations are a generalized trait linked to vocalizations emitted in dangerous situations, we expected to find fast periodicities in bat distress calls. Bat distress calls are typically short, lasting on average less than 10 ms^24^. Since amplitude modulation cycles have to fit within the sounds’ duration, we reasoned that any form of roughness found in bat distress calls should be much faster than that observed in human screams. The data corroborated our hypotheses. We show that there exists a form of acoustic roughness in bat distress vocalizations and that rough-like sounds entrain field potentials and are more effective in accelerating the heart rate of listening bats than slow amplitude modulated sounds. We also describe that, as suspected, temporal modulations found in bat distress calls are superfast, reaching the order of kHz (i.e. bat roughness occurs at ~1.7 kHz vs. 30–150 Hz in humans). Albeit large interspecific differences between bats and humans, our findings speak in favor of fast temporal modulations in the sounds uttered as generalized trait capturing the listeners’ attention while the emitter is under duress.

## Results

### Fast amplitude modulations are present in bat distress vocalizations

We recorded distress calls from 13 adult bats (6 females and 7 males) of the species *C. perspicillata*. This species emits sequences of distress calls composed of basic vocalization units defined as “syllables”^24^. In bats, the production of distress calls can be triggered by holding the animals in the hands while carefully caressing the neck-skin^22,23^.

**Figure 1:**
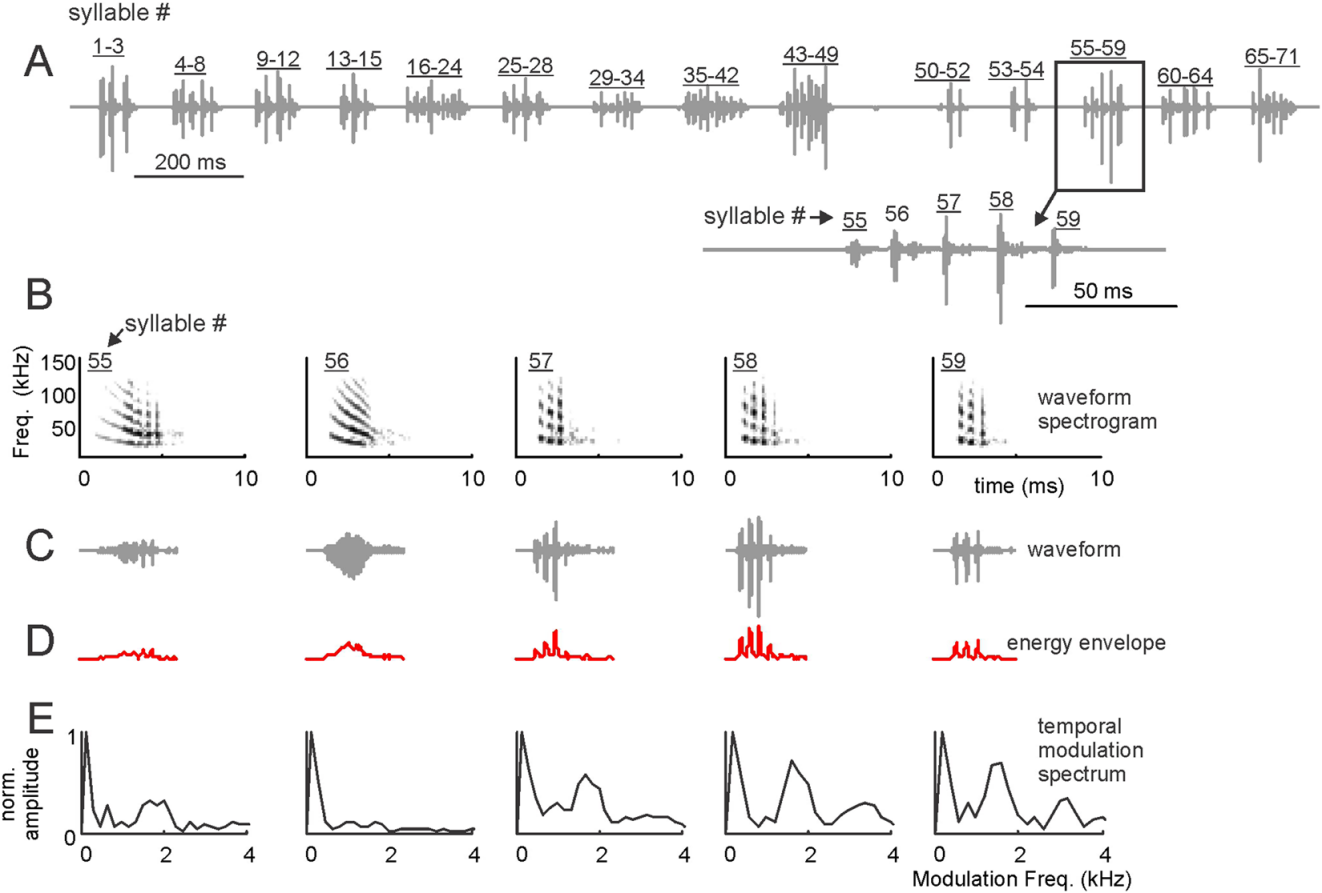
Bat distress vocalizations carry superfast periodicities. (**A**) Example distress sequence containing 14 syllable groups (bouts) and 71 syllables. A zoom-in into the bout composed of syllables 55–59 is provided. (**B**) spectrograms, (**C**) time waveforms, (**D**) envelope and (**E**) temporal modulation spectra (TMS) of syllables 55–59. Note that fast periodicities at ~1.7 kHz occur in syllables 55, 57–59, but are less clear in syllable 56. Also note that all example distress syllables shown are harmonically-structured downward frequency modulated sweeps.

We studied a total of 114 distress “sequences”. Each of those sequences was composed of sound units defined as “syllables” (see ref. ^24^. for the definition of distress sequences and syllables). An example distress sequence is shown in Fig. 1A. This sequence contained 71 syllables arranged over a time period of 2.38 s. As shown in Fig. 1A and in a previous article^24^, within a distress sequence, syllables are temporally arranged in groups defined as “multi-syllabic bouts”. A zoom-in into the multi-syllabic bout containing syllables 55–59 is shown at the bottom of Fig. 1A to illustrate the temporal separation between syllables.

We searched for fast, periodic amplitude fluctuations (i.e. roughness-like patterns) in individual distress syllables (Fig. 1B–E). To that end, the energy envelope of each syllable was calculated (Fig. 1D) and the spectrum of the envelope (defined as the temporal modulation spectrum, TMS, Fig. 1E) was obtained and analyzed in the range between 0–4 kHz. As it can be noted in the example distress syllables represented in Fig. 1B–E, a single distress sequence could contain syllables with different types of TMS. For example, the TMS of syllables 55, 57, 58 and 59 (Fig. 1B–E) had a pronounced peak at ~1.7 kHz. In syllable 56, the peak at ~1.7 kHz was less evident. We reasoned that syllables modulated at rates ~1.7 kHz could represent rough-like sounds in bats, since they contained a pronounced temporal modulation pattern, the hallmark feature of acoustic roughness, at least in humans^1^. Note that 1.7 kHz is a very low frequency for *C. perspicillata*, a bat species that can reach frequencies above 100 kHz both while echolocating and while producing communication calls^24,34,35^. In fact, the cochlear frequency response curves of *C. perspicillata*, calculated using distortion product otoacoustic emissions, suggest that hearing in this animal species deteriorates at frequencies below 5 kHz (see upcoming text in the results section).

**Figure 2:**
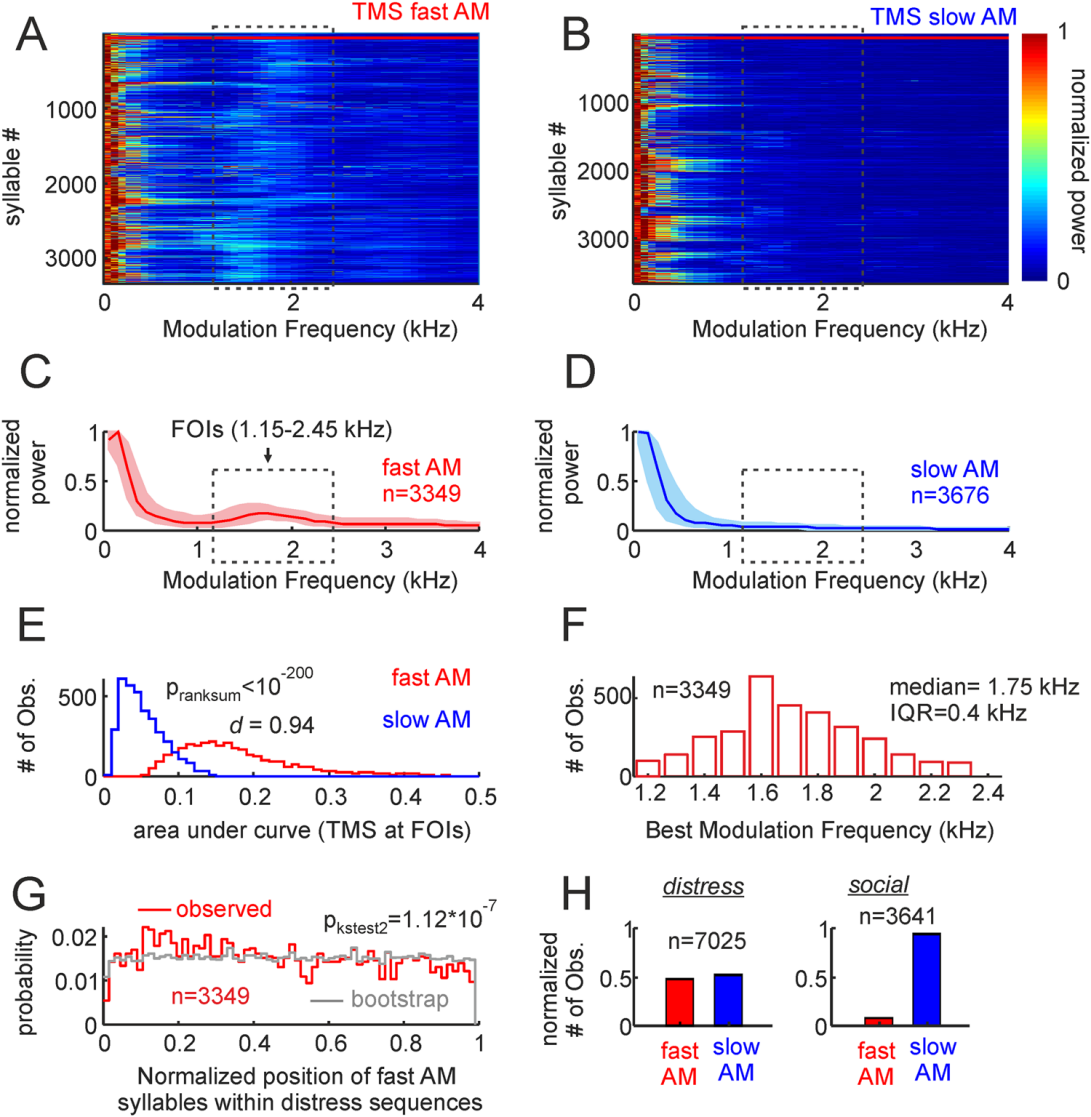
Temporal properties of fast and slow amplitude modulated (AM) vocalizations. (**A**,**B**) Show the TMS of the two syllable groups, represented as colormaps. The first 50 fast AM and slow AM calls in the colormaps (border marked by horizonal red lines) were used to train the support vector machine classifier (see also supplementary Figure S1). (**C**,**D**) Are median TMS of all fast and slow AM calls studied (25th and 75th percentiles shown as shaded areas). Note that a peak occurs at the frequencies of interest (FOIs) in fast AM calls but not in slow AM vocalizations. (**E**) Histogram of the area under the TMS curve at the FOIs in the two call groups. The p-value of a Wilcoxon ranksum test and the d size-effect metric are provided. (**F**) histogram of best modulation frequencies found in the population of fast AM calls. Median and inter-quartile range are provided. (**G**) Probability of finding fast AM vocalizations in certain positions along the distress sequences. To calculate probability values, the relative position of each fast AM call was obtained taking into account the length of the sequence in which it occurred. This “observed” probability distribution was compared (Kolmogorov-Smirnov two-sample test) with an “expected” distribution obtained by randomly swapping the position of syllables within each sequence 100 times. (**H**) The percentage of fast and slow AM calls found when bats where under duress (distress) and when interacting between them in a keeping cage (social, see also Supplementary Fig. S3).

We classified the distress syllables recorded into fast amplitude modulated vocalizations (fAMVs) and slow amplitude modulated vocalizations (sAMVs) based on their TMS. For that purpose, we relied on a binary support vector machine (SVM) classification algorithm that was fed with the TMS of all 7025 distress syllables recorded. The SVM classifier was trained with two sets of TMSs comprising the TMS of 50 fAMVs and 50 sAMVs (see training TMS sets in Supplementary Fig. S1). The results obtained with the SVM classifier are depicted in Fig. 2. Altogether, 3349 out of 7025 syllables studied (47.7%) were classified as fAMVs. The TMS of all fAMVs and sAMVs are shown as colormaps in Fig. 2A,B, respectively. Note that in the range from 1.15 to 2.45 kHz, brighter colors are present in the population of fAMVs when compared to sAMVs. This range is marked by a rectangle in Fig. 2A,B and it was defined as the “Frequencies of Interest” (FOIs) for further analysis. The presence of high energy at the FOIs was also visible in median curves for the populations of fAMVs and sAMVs (Fig. 2C,D) identified by the SVM classifier.

To validate amplitude modulation differences at the population level, we calculated the area under the curve at the FOIs in the two syllable groups (Fig. 2E). As expected, the power at the FOIs was significantly higher in fAMVs than in sAMVs (Wilcoxon ranksum test, p < 10^−200^) and this differences had a large effect size (Cliff’s delta (*d*)= 0.94, following^36^: negligible effect: absolute *d* value (abs (*d*)) < 0.147, small: 0.147 < abs(*d*) < 0.33, medium: 0.33 < abs(*d*) < 0.474, large: abs(*d*) > 0.474). Note that the statistical analysis described in the preceding text was conducted by pooling together data from all fAMVs and sAMVs recorded across animals and distress sequences. Strong differences regarding amplitude modulation were also observed when comparing the median power at FOIs in fAMVs and sAMVs recorded within the same distress sequences using paired statistics (*Signrank*, p = 3.6*10^−15^, see Supplementary Fig. S2A).

The best modulation frequency (BMF) of each fAMV was calculated by searching for the frequency that contained the highest energy in the FOI range (that is, between 1.15 and 2.45 kHz). The BMF distribution had a median of 1.75 kHz with an interquartile range (IQR) of 0.4 kHz (Fig. 2F). To determine if fAMVs occurred at a preferred position within distress sequences, the normalized position of each fAMV was calculated relative to the length of the sequence in which it occurred. Though fAMVs occurred throughout the sequences, the distribution of preferred positions was slightly skewed to the left (Fig. 2G, median = 0.45, IQR = 0.49). The latter points towards a higher probability of finding rough-like syllables in the first half of the distress sequences. This trend was validated statistically by comparing the temporal syllable distribution observed to a bootstrap distribution created by randomizing the positions of fAMVs and sAMVs in each sequence (100 randomizations for each sequence, two-sample Kolmogorov-Smirnov test: p = 1.12*10^−7^, Fig. 2G). Comparing the probability of finding fAMVs in the first and second sequence halves also indicated statistical significance (*Signrank test*, p = 0.03).

### Fast amplitude modulation could be a hallmark feature of bat distress calls

The results presented thus far in this article demonstrate the occurrence of fast amplitude modulation ~1.7 kHz in 47.7% of the distress syllables studied. However, if fast amplitude fluctuations are a hallmark of distress calling, then the percentage of fAMVs should be much lower in other types of social vocalizations. To test this idea, we studied vocalizations of the same 13 bats in which distress calls were studied but, in this case, when the animals were interacting in a keeping cage. We reasoned that bats that are accustomed to each other (they were placed together for an entire week before the recordings took place) should not engage often in agonistic interactions that could involve the production of distress-like sounds. Note that acoustic recordings obtained in this broad “social” context cover many types of interactions between bats and we cannot link each sound recorded to specific behavioral contexts as only acoustic data was collected.

Altogether, we recorded 3641 vocalizations in the “social” context (echolocation calls were excluded based on their spectral design). The same SVM classifier used to split distress calls into fAMvs and sAMVs was used to classify social calls. Supplementary Fig. S3 shows the TMS and spectra of all social calls studied based on their classification as fAMVs and sAMVs. The number of social calls classified as fAMs amounted to 229, representing 6.3% of the total number syllables studied in the social context (3641 syllables). This value is much lower than the value obtained during distress calling (47.7%, see Fig. 2H) suggesting that the occurrence of fast amplitude modulations could indeed be a hallmark of distress calling in bats.

### Fast and slow amplitude modulated distress vocalizations differ in their peak frequency and bandwidth

For the remaining acoustic analysis presented in this manuscript we will focus only on vocalizations studied in the distress calling context (i.e. handheld bats while massaging the neck skin).

**Figure 3:**
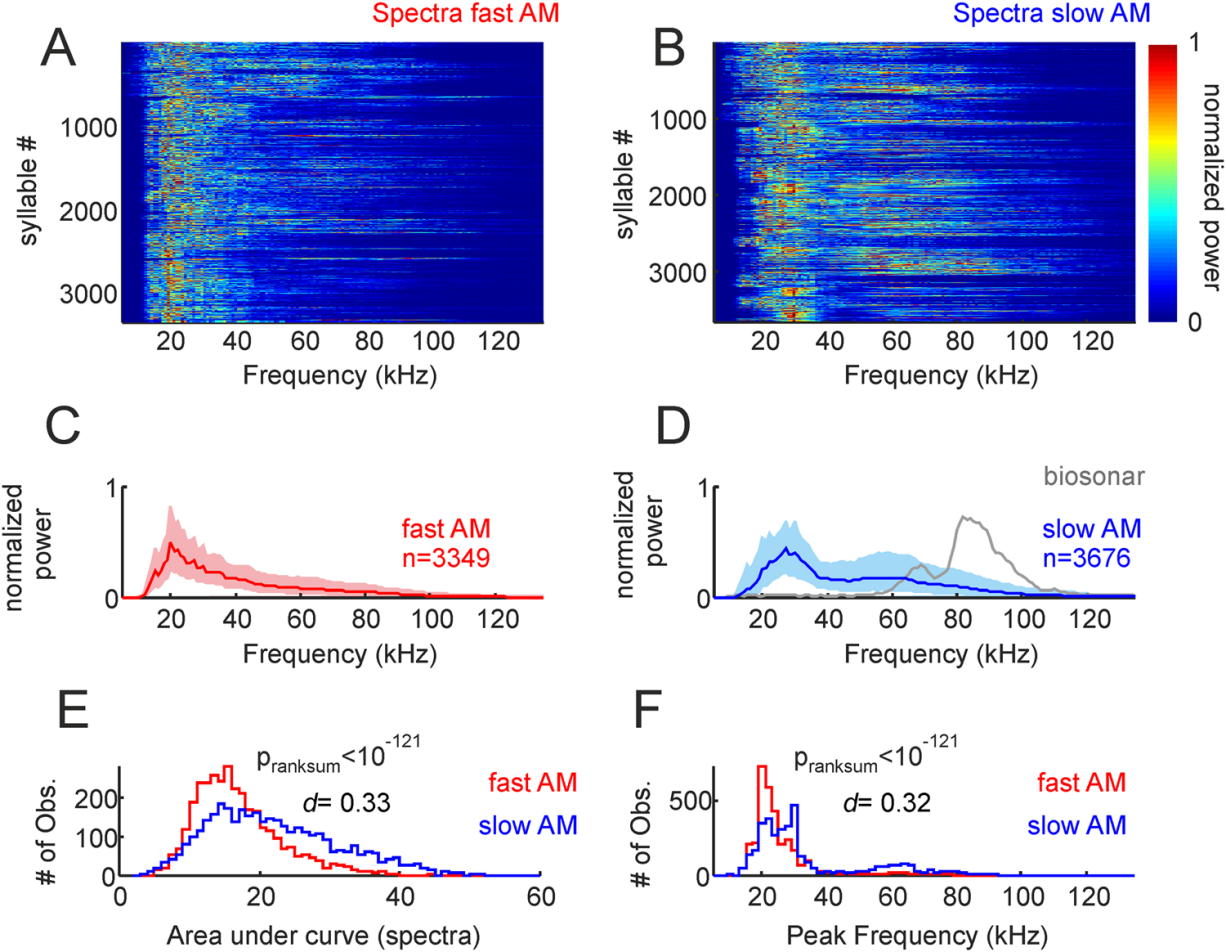
Spectral properties of fast and slow amplitude modulated (AM) vocalizations. (**A** and **B)** Show the spectra of the two syllable groups, represented as colormaps. (**C** and **D**) Are median spectra of all fast and slow AM calls studied (25th and 75th percentiles shown as shaded areas). The median spectrum of 100 biosonar calls is shown in D for comparison purposes. (**E**) Histogram of area under curve calculated from the spectra of fast and slow AM vocalizations. The p-value of a Wilcoxon ranksum test and the d size-effect metric are provided. (**F**) Histogram of peak frequency in fast and slow AM calls.

We tested for spectral differences between fAMVs and sAMVs produced during distress calling. At the population level, there was a tendency for fAMVs to have a narrower spectrum than sAMVs, with fAMVs tending to have higher power in the range from 40–80 kHz. The latter is visible in both the normalized spectra of all fAMVs and sAMVs (colormaps in Fig. 3A,B) and in the median spectra of the two syllable groups (Fig. 3C,D). Differences in spectral bandwidth between the two syllable groups were statistically significant, as validated by a ranksum test that compared the area under the normalized spectra in fAMVs and sAMVs (Fig. 3E, p_ranksum_ < 10^−121^). Note that the *d*-metric obtained for this comparison indicated a medium/small size effect (*d* = 0.33). Besides these small differences in spectral bandwidth, fAMVs and sAMVs also differed in their peak frequencies (Fig. 3F, median_fAMVs_ = 22 kHz_,_ median_sAMVs_ = 27 kHz, p_ranksum_ = 10^−121^) with fAMVs tending to have lower peak frequency values, although the size effect in this case was also small (*d* = 0.32). Note that spectral differences between fAMVs and sAMVs were also apparent when comparing median bandwidth and peak frequency values obtained within the same distress sequences using paired statistics (see Supplementary Fig. S2B,C, peak frequency, *Signrank*, p = 1.6*10^−6^, bandwidth, *Signrank*, p = 4.7*10^−4^).

Our data allows to assess how much bats separate carrier and modulating waves during natural calling, since we measured peak frequency (i.e. the carrier with the strongest energy expressed in Hz) and BMF (frequency of modulating wave, also in Hz) of each distress syllable classified as fAMV. To that end, we calculated carrier/modulator ratios by dividing the peak frequency of each fAMV by its BMF. The data indicated that fAMVs produced during distress calling have median carrier/modulator ratios of 15.2 (IQR = 5.0), in other words, in rough-like sounds (fAMVs) produced by bats the modulating wave is well separated from the most energetic carrier.

### Fast and slow amplitude modulated distress vocalizations differ in their spectral smoothness and regularity

**Figure 4:**
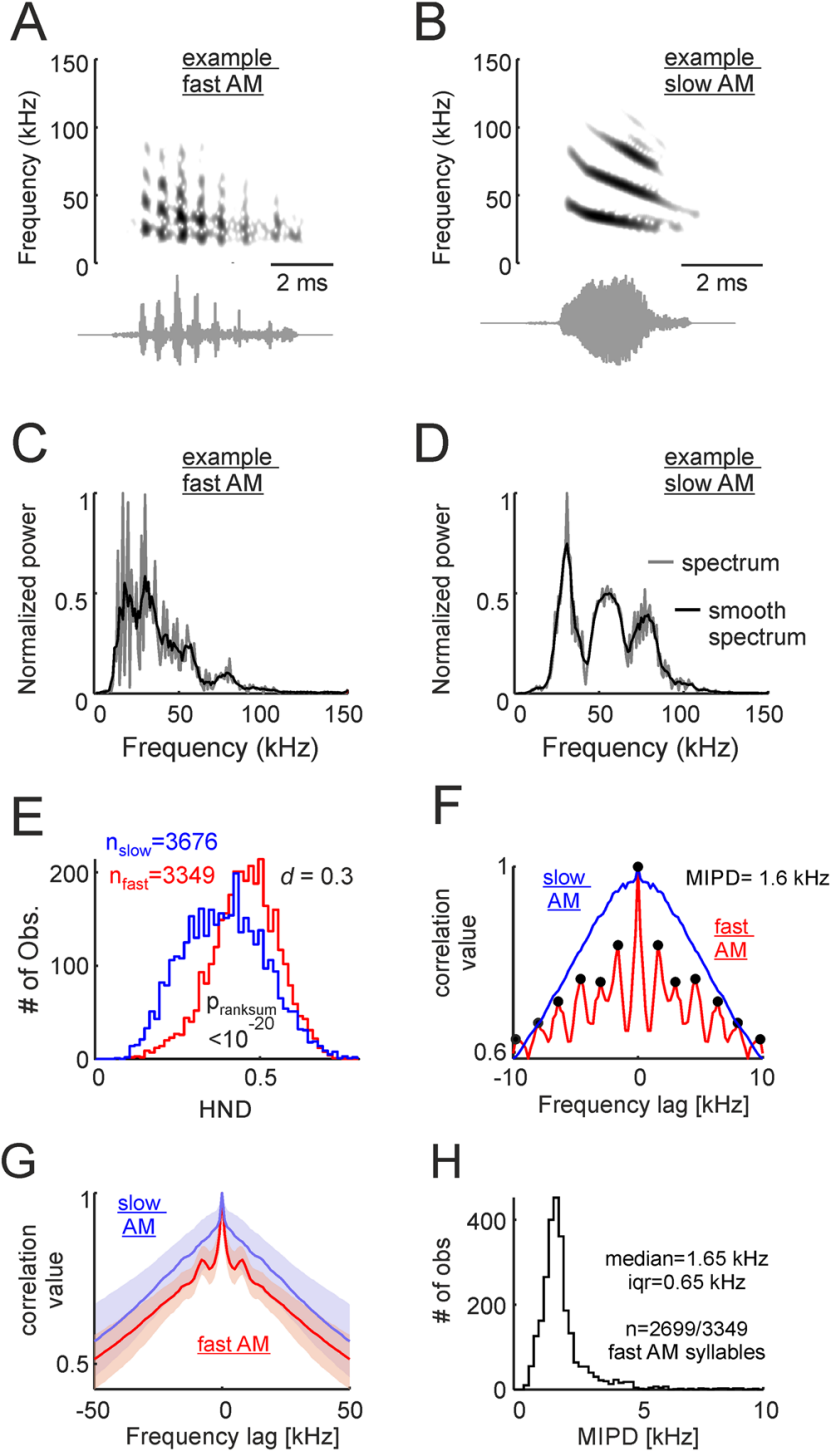
Spectral regularities are a characteristic feature of fast amplitude modulated vocalizations (fAMVs). (**A** and **B**) Show the spectrograms and waveforms of one example fAMV and one slow modulated vocalization (sAMV, A and B, respectively). (**C** and **D**) Are the spectra of the same two example vocalizations. Note that the observed spectra (200-Hz frequency resolution) and “smooth” spectra are represented. Smooth spectra were calculated using a 5-point moving average. The difference between the observed and the smooth-spectra was used for harmonic-to-noise difference (HND) calculations. (**E**) Histograms of HND for fAMVs and sAMVs. The p-value of a Wilcoxon ranksum test and the d size-effect metric are provided. (**F**) Spectral autocorrelograms for the example fAMV and sAMV shown in (**C** and **D**). Note that spectral regularities (i.e. peaks spaced at regular distances) occurred in the example fAMV. In this example, the median inter-peak distance (MPID) was 1.6 kHz. (**G**) Median spectral autocorrelograms for all fAMVs and sAMVs studied (25th and 75th percentiles shown as shaded areas). Note the side- peaks occurring in the population of fAMVs. (**H**) Histogram of MPID for the fAMVs in which more than one peak could be detected in the spectral autocorrelogram. Median and interquartile range are (iqr) given.

There were also differences between distress fAMVs and sAMVs regarding their harmonic-to-noise difference (HND, Fig. 4). The HND-metric is useful for quantifying spectral “smoothness”, and it is calculated as the difference between the observed spectrum and the same spectrum smoothened using a moving average filter (here a 5 point moving window applied to spectra calculated with 200-Hz frequency resolution; see Fig. 4A–D for illustration of the HND calculation in one SAMV (Fig. 4A,C) and one sAMV (Fig. 4B,D)). This method was originally proposed for studying “hoarseness” in human speech^37^ and it has since been used in several studies on vocalizations produced by humans and other animal species (e.g. dog barks^38^). Calculating the HND of fAMVs and sAMVs produced by *C. perspicillata* rendered statistical differences between the two syllable groups (Fig. 4E, p_ranksum_ = 10^−20^, median_fAMVs_ = 0.45_,_ median_sAMVs_ = 0.39, small effect size (*d* = 0.3)) thus indicating that the spectra of rough-like syllables (fAMVs) is less smooth than that of slow modulated syllables (sAMVs). This effect was also observable when comparing median HNDs obtained within the same distress sequences using paired statistics (see Supplementary Fig. S2D, *Signrank test*, p = 0.02).

Note that the fact that fAMVs had the least smooth spectra does not imply that their spectra were “irregular”. In fact, we observed that in the spectra of fAMVs peaks occurred at regular intervals. The latter is illustrated in the spectrum autocorrelation function represented in Fig. 4F for one example fAMV (same as in Fig. 4A,C). In this example autocorrelogram, local peaks (Matlab findpeaks function, peak prominence 0.025) could be detected every 1.6 kHz (i.e. 8 samples of the autocorrelogram of a 200-Hz resolution spectrum). Side-peaks indicating spectral regularity were also observed in the median autocorrelogram of all syllables labeled as fAMVs by the SVM classifier, but not in the median autocorrelogram of syllables labeled as sAMVs (Fig. 4G). The local-peak detection algorithm rendered more than 1 peak in 2699 out of 3349 syllables identified as fAMVs (80.6%). In those syllables, we calculated the mean inter-peak distance (MIPD) from the autocorrelogram as a metric of spectral regularity. The MIPD distribution peaked at 1.6 kHz (Fig. 4H). Note that this value is close to best amplitude modulation frequency values determined by analyzing the temporal modulation spectrum (i.e. 1.75 kHz for all fAMVs, see Fig. 3, but 1.65 kHz for the 2699 fAMVs in which the MIPD could be measured). In fact, paired statistics comparing MIPD and best amplitude modulation frequencies of sAMVs rendered no statistically significant differences (p_signrank_ = 0.49). The latter suggests that temporal and spectral modulations are strongly linked to each other, a situation that is expected if one considers the spectral regularities observed as “sidebands” created by the presence of a modulating wave.

### 1.7 kHz is absent as carrier frequency in fast amplitude modulated vocalizations

**Figure 5:**
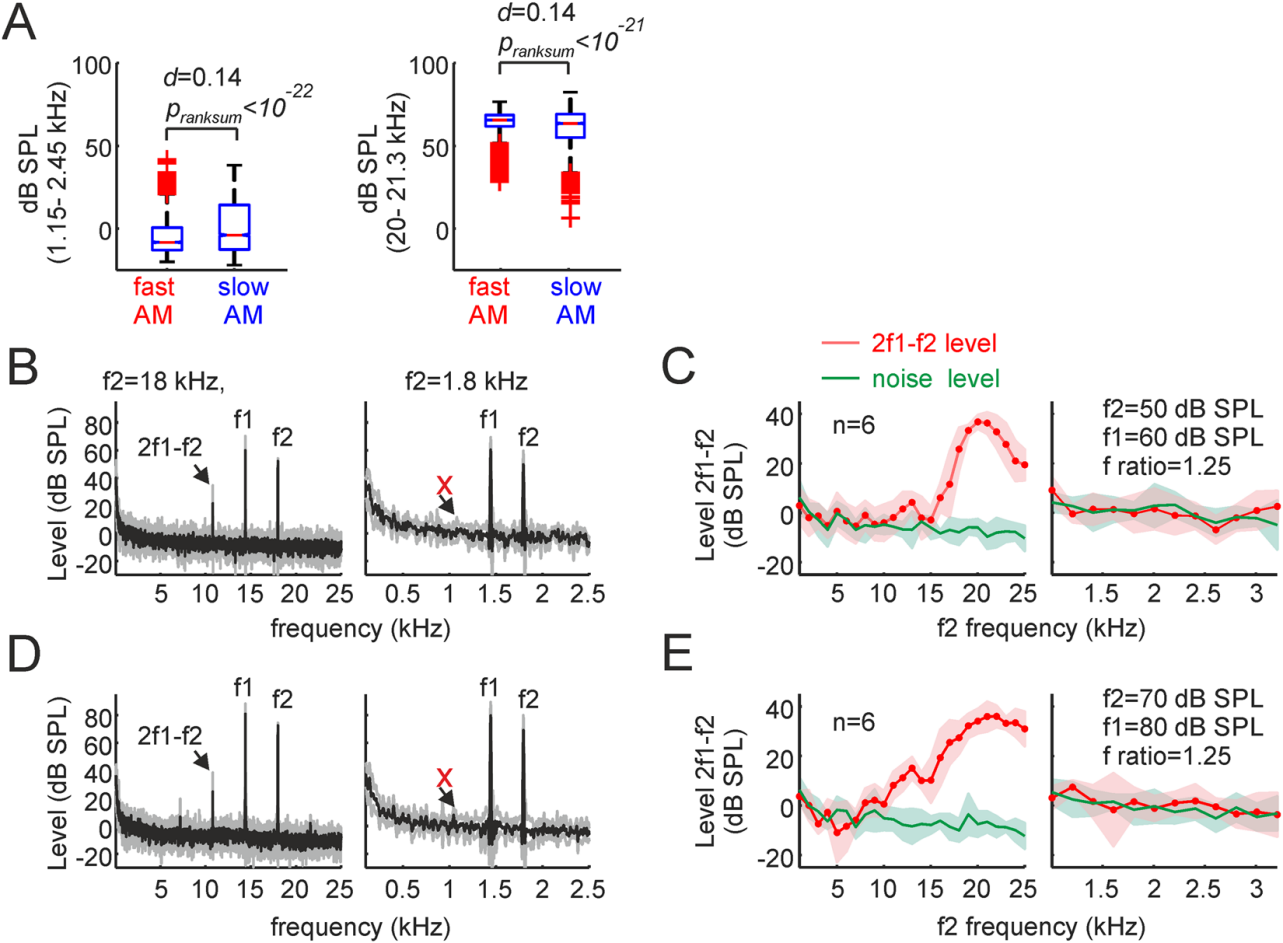
1.7 kHz is has low power as carrier frequency and does not trigger responses in the bat cochlea. (**A**, left) Sound pressure level (in dB SPL) calculated in the range from 1.1–2.5 kHz. Note that this frequency range was not well represented in the sounds uttered, i.e. values were always below 50 dB SPL in fast modulated and slow modulated vocalizations, and median SPLs were close to 0. The p-value of a Wilcoxon ranksum test and the d size-effect metric are provided. The same analysis is shown in the right panel of A for the frequency range between 20–21.3 kHz for comparison purposes. On each box, the central mark indicates the median, and the bottom and top edges of the box indicate the 25th and 75th percentiles, respectively. The whiskers extend to the most extreme data points not considered outliers, and the outliers are plotted individually using the ‘+’ symbol. (**B**) Average distortion product (DP) measurements obtained in six animals for f2 frequencies of 18 kHz and 1.8 kHz (left and right, respectively), when the f2 level was set to 50 dB SPL. Note that a strong cubic DP (2f2-f1) appeared when f2 was equal to18 kHz, but not when it was 1.8 kHz. (**C**) Coarse and high-resolution DPgrams obtained with frequency steps of 1 kHz and 200 Hz (left and right, respectively). Coarse DPgrams covered f2 frequencies from 1–25 kHz, while high-resolution DPgrams covered f2 frequencies from 1- 3.2 kHz. In high-resolution DPgrams, the DP level measured remained within the acoustic noise level, thus indicating poor hearing sensitivity for those frequencies. (**D**,**E**) Show DP measurements similar to those depicted in B and C, but for stimulus levels of 80/70 dB SPL.

We have shown that almost half of the bat distress syllables carry periodicities (roughness-like patterns) at frequencies ~1.7 kHz. Such periodicities can be measured in both the time and spectral domains and could be interpreted as the syllables’ modulating frequency. We tested whether 1.7 kHz was missing or present as carrier in the syllables’ spectra. The latter was achieved by measuring the level (in dB SPL) in the range between 1.15 kHz–2.45 kHz (the FOIs) in the frequency spectrum of each syllable (not in its TMS). The level in this frequency range was obtained by computing the logarithm of the root-mean-square (RMS) of the filtered signals in the FOI range (3^rd^ order Butterworth filter), and by comparing the results with the RMS of a 94 dB SPL pure tone (1 kHz) produced by a calibrator (see methods). Overall, the level in the FOI range never exceeded 50 dB SPL, regardless of whether it was studied in fAMVs or sAMVs (Fig. 5A). The average FOI level for fAMVs was −3.9 dB SPL while for sAMVs the average level reached the 0.6 dB SPL. FOI level values were significantly higher in sAMVs than in fAMVs (p_ranksum_ < 10^−22^) although the size effect of this comparison indicated negligible effects (*d* = 0.14). Note that the level values obtained in the FOI range were much lower than those obtained in the range from 20–21.3 kHz (mean level fAMVs = 64.4 dB SPL, mean level sAMVs = 61.2 dB SPL, p_ranksum_ < 10^−21^, *d* = 0.14). Overall, the low sound pressure levels observed when syllables were filtered in the FOI range suggest that 1.7 kHz is not a carrier frequency but rather a modulating wave responsible for the temporal and spectral regularities measured in rough-like distress syllables.

### The bat cochlea does not respond to carriers ~1.7 kHz based on non-linear mechanics

We also tested whether the bats’ ears were sensitive to carrier frequencies ~1.7 kHz (the putative modulator of fAMVs). This was a necessary test, because we noticed that previous studies on *C. perspicillata’s* audiogram always measured hearing sensitivity at frequencies above 5 kHz^39–41^. We measured the cochlear audiogram of 6 adult awake *C. perspicillata* (3 males, 3 females) by means of distortion product otoacoustic emissions (DPOAE). DPOAEs are a by-product of nonlinear ear mechanics and they represent a non-invasive objective method for measuring sensitivity and tuning of the cochlear amplifier^42–44^. We focused on the cubic DPOAEs that occur at frequencies of 2f1-f2, were f2 and f1 represent the frequencies of two sounds produced simultaneously by loudspeakers placed closed to the bats’ tympanic membrane. The ratio between f1 and f2 was kept constant at 1.25 and there was a level difference of 10 dB between the sounds to optimize stimulus parameters (see refs. ^44,45^). To test for the occurrence of DPOAEs, two stimulus level combinations were used (L1/L2 = 50/60, 80/70 dB SPL). DPOAEs were measured with coarse and fine frequency steps, covering f2 frequencies between 1–25 kHz (steps of 1 kHz) and between 1–3.2 kHz (steps of 200 Hz), respectively.

As it can be seen in Fig. 5B,D, when f2/f1 sound pairs of 18/14.4 kHz were presented, a noticeable cubic distortion appeared, regardless of whether f2 was presented at 50 or 70 dB SPL (Fig. 5B,D (leftmost panels), respectively). However, no visible distortion product occurred in response to f2/f1 pairs of 1.8 and 1.44 kHz, regardless of the f2 level tested (Fig. 5B,D (rightmost panels)). Overall, high amplitude distortion products, indicating strong cochlear amplification, were visible only for f2 frequencies above 5 kHz (Fig. 5C,E, leftmost panels). In response to lower f2 frequencies, distortion product amplitude fell within the acoustic noise level (Fig. 5C,E, rightmost panels). These results indicate that *C. perspicillata’s* cochlea is not well suited for dealing with faint low frequency sounds and can therefore not respond to potential 1.7 kHz carrier frequencies of fAMVs even if those frequencies were more intense than 60 dB SPL, which is not the case according to our data (see SPL values in Fig. 5A).

### The modulation power spectrum of distress syllables

**Figure 6:**
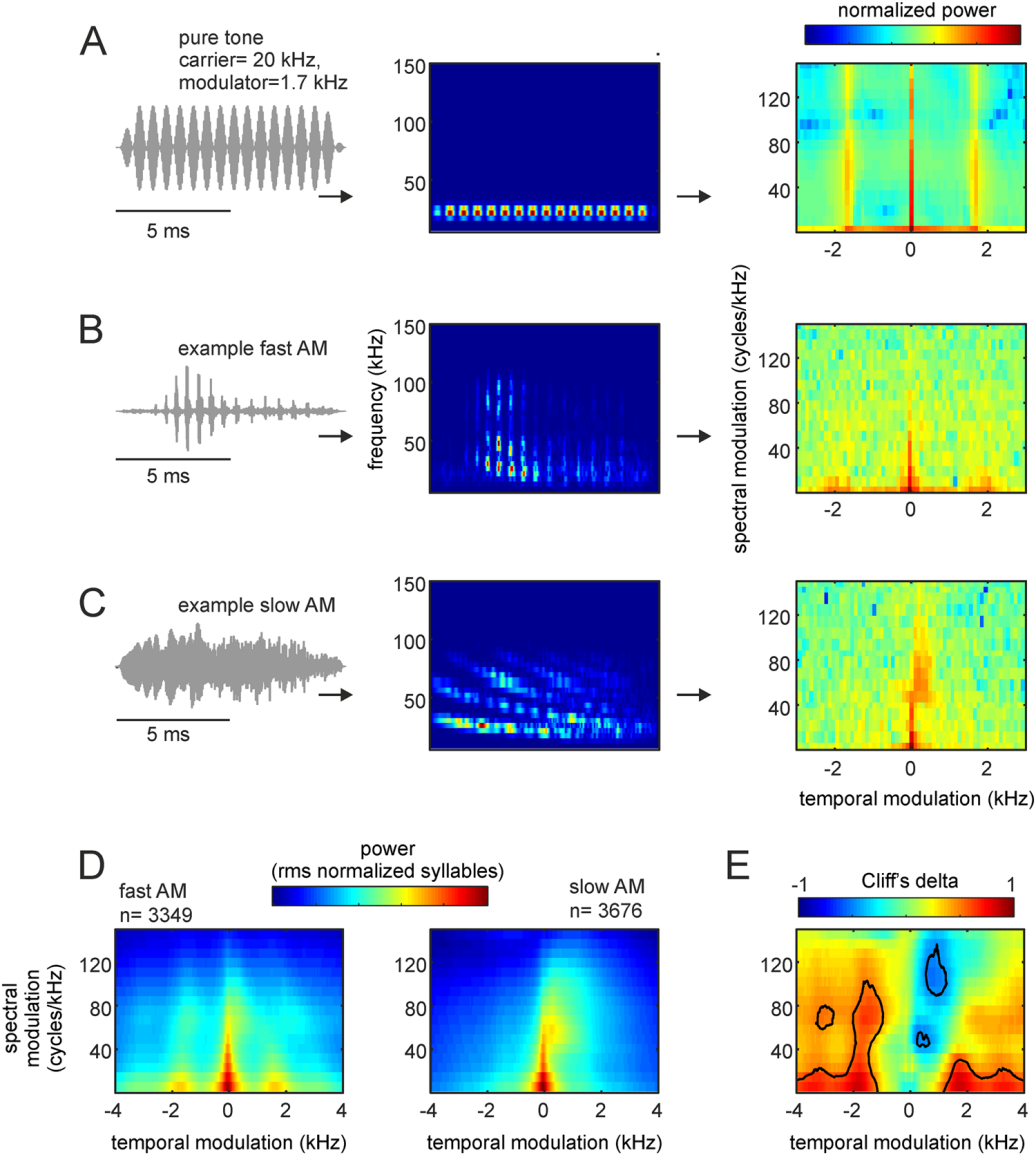
Modulation power spectra (MPS) of bat distress vocalizations. (**A**, from left to right) The waveform, spectrogram, and MPS of a pure tone temporally modulated at 1.7 kHz. Note that the temporal modulation appears in the MPS as side-peaks at −1.7 and 1.7 kHz. (**B**,**C**) show the waveform, spectrogram, and MPS for an example fast and one slow amplitude modulated (AM) vocalization, respectively. Note that fast temporal modulations are visible in the modulation power spectrum of the example fast AM vocalization. (**D**) Average MPS for the population of fast and slow AM calls studied. (**E**) Cliff’s delta (d) obtained after comparing the MPS of the syllable groups. Contour lines indicate large size-effects (i.e. d > 0.478), indicating consistent MPS differences.

The modulation power spectrum (MPS) of fAMVs and sAMVs was calculated (Fig. 6). The MPS is calculated from the 2D fast Fourier transform (FFT) of the syllables’ spectrogram (see below and methods). The MPS represents power in the two-dimensional space of temporal and spectral modulations and it has been used to study vocalizations in other highly vocal animal groups such as humans and birds^1,46,47^.

We were interested in the MPS because of two reasons: (i) unlike classical acoustic analysis techniques (as those described in the preceding text), the MPS allows to quantify amplitude and spectral modulations simultaneously in each syllable^47^; and (ii) filtering the MPS provides a robust technique for removing modulation components of the signal without changing other signal attributes. In our case, we were interested in determining whether the presence/absence of fast amplitude fluctuations (putative roughness) had differential effects on the bats’ heart rate and neural responses (see below).

The oscillogram, spectrogram, and MPS of a 20 kHz pure tone modulated at 1.7 kHz, one example fAMV, and one example sAMV are shown in Fig. 6A–C, respectively. It can be noted that in both the amplitude modulated pure tone and the example fAMV, more power occurred at temporal modulation frequencies close to 1.7 kHz. In the MPS, temporal modulations are represented in the positive and negative planes, with the former indicating the presence of downward frequency modulations and the latter corresponding to upward frequency modulations^46,47^. The presence of strong downward spectral modulations (hence positive values in the temporal modulation domain) is noticeable in the example sAMV represented Fig. 6C. This downward spectral modulation was strongest in the range between 50–80 kHz, corresponding to the call’s bandwidth.

As expected, pronounced power at temporal modulation frequencies close to 1.7 kHz was also evident when averaging MPS curves of all distress syllables classified as fAMVs (n = 3349), but not in the average MPS of sAMVs (n = 3676, Fig. 6D). We calculated Cliff’s delta to assess the effect size of differences between the MPS of fAMVs and sAMVs (Fig. 6E). The comparison between the two syllable types was done for each temporal- and spectral-modulation combination in the MPSs. As mentioned in the preceding text, *d* values above 0.478 were considered as large effect size (contour lines in Fig. 6E) following previous studies^36^. Overall, the values obtained from *d* calculations validated the existence of two main MPS differences between fAMVs and sAMVs: (i) faster temporal modulations in fAMVs than in sAMVs, and (ii) more pronounced downward spectral modulations in sAMVs.

### Testing the effect of fast amplitude modulations on the listeners’ physiology

The acoustic analysis described in the previous sections revealed the presence of fast amplitude modulation (putative roughness) in 3349 out of 7025 syllables studied (47.7%). To determine whether bats could actually perceive fast amplitude fluctuations at 1.7 kHz we measured the heart rate (HR) response of awake animals while they listened to sequences of natural fAMVs and their demodulated versions. Previous studies have shown that the bats’ HR increases when the animals are subject to fear conditioning, when they listen to aggression (versus non-aggression) calls, or after electric stimulation of the amygdala^31,48,49^. We thus reasoned that the HR could be a useful indicator of autonomic changes driven by the presence of fast amplitude modulations in the sounds.

**Figure 7:**
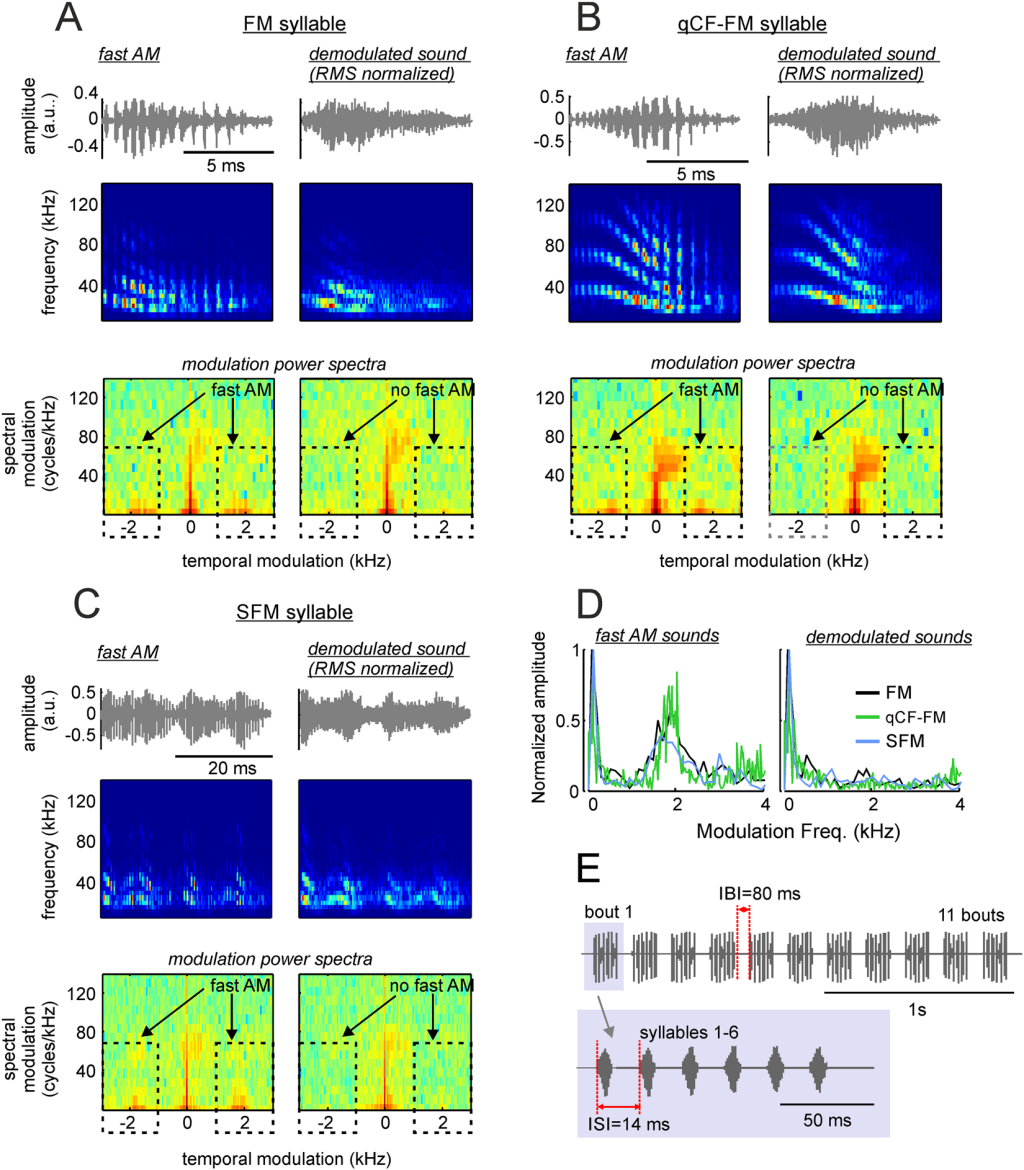
Removing fast periodicities by filtering modulation power spectra (MPS) of fast amplitude modulated (AM) vocalizations. (**A)** Left column shows the waveform, spectrogram, and MPS of a downward frequency modulated (FM) syllable containing fast periodicities. (**A**) Right column shows representations of the same sound after demodulation by filtering the MPS in the range marked with the dashed lines (see methods and Supplementary Fig. S4). (**B,C)** show the same representations as A, but for one syllable containing quasiconstant-frequency and downward frequency modulated components (qCF-FM syllable, **B**) and one syllable composed of sinusoidal frequency modulations (SFM syllable, **C**). The three syllables represented in (**A–C)** and their demodulated treatments were used as stimuli for measuring electrocardiogram (ECG) and neural responses. (**D**) The temporal modulation spectra of the FM, qCF-FM, and SFM syllables (left) and their demodulated treatments (right). (**E**) Temporal arrangement of syllables in the sequences used as stimuli during ECG and neural measurement experiments. Altogether, six sequences were constructed, each composed of the same syllable repeated at inter-syllable intervals (ISIs) of 14 ms. The syllables formed 11 bouts that were repeated at inter-bout intervals (IBIs) of 80 ms.

To determine whether roughness had specific effects on the bats’ HR the MPS of three natural fAMVs was filtered to produce their “demodulated” versions (Fig. 7). Using natural sAMVs as control also could have been an option. We did not choose this option because natural vocalizations always differ between them in more than one acoustic parameter (even if in a subtle manner unperceivable to us). We reasoned that multi-parametric differences between sounds that are not under precise control of the experimenter could have hampered the interpretation of the results obtained.

As stimuli for sound demodulation we chose one frequency modulated (FM) syllable (Fig. 7A), one syllable containing quasiconstant frequency and FM components (qCF-FM, Fig. 7B), and one containing sinusoidal frequency modulations (SFM, Fig. 7C). Note that in a previous study we reported that syllables containing downward FM components represent 94% of the distress vocalizations produced by *C. perspicillata*, SFMs (the next best represented group) amounted to ~4% of the syllables analyzed, while qCF syllables represented less than 1% of the syllables studied^24^.

The procedure used for MPS filtering was designed according to previous studies in humans^47^ and is described in detail in the methods section and in the Supplementary Fig. S4, which shows the demodulation procedure for one of the sounds used as stimuli. MPS filtering allows to modify certain features of the vocalizations without affecting others. Here, we used MPS filtering for removing the syllables’ putative roughness occurring at ~1.7 kHz without changing their spectro-temporal structure (see spectrograms in Fig. 7A–C). The result (Fig. 7A–D) was three pairs of natural fAMVs and software-demodulated syllables (artificial sAMVs) that were used as stimuli for measuring HR responses. Note that the TMS of artificial sAMVs produced after MPS filtering resembles that of natural sAMVs produced by the bats (Fig. 7D).

**Figure 8:**
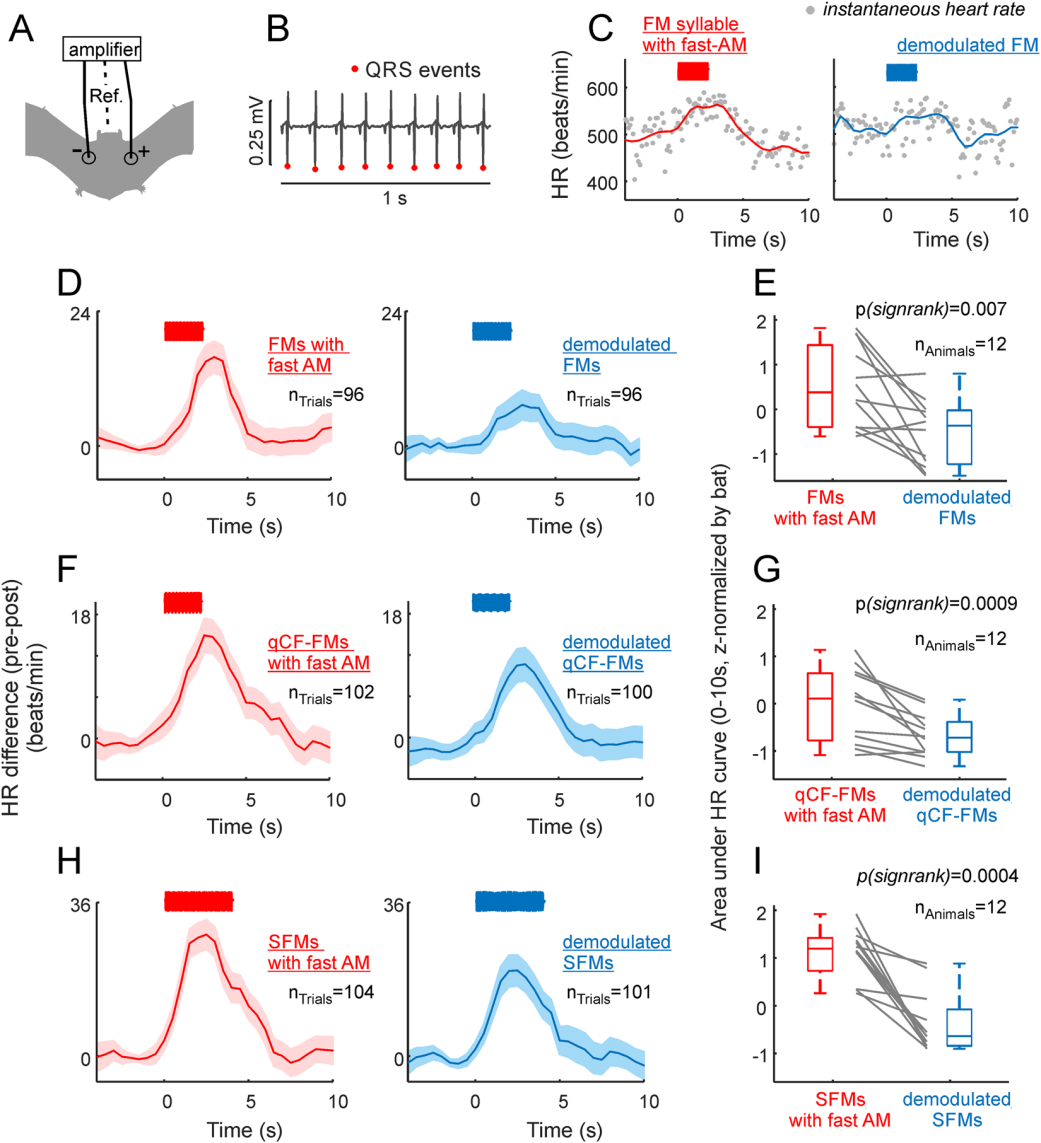
The presence of fast amplitude modulation boosts the bats’ heart rate (HR). (**A**) Schematic representation of electrode positioning during electrocardiogram (ECG) measurements. (**B**) 1s-segment of ECG recording. The position of QRS events is indicated. (**C**) Instantaneous HR in two recording trials in which the fast amplitude modulated (AM) FM syllable and its demodulated treatment were used as stimuli (left and right, respectively). HR-curves (solid-lines) were obtained by interpolation from the instantaneous HR data. (**D**) Average HR curves obtained considering all presentations of the fast-AM FM syllable (left panel) and its demodulated treatment (right panel). Shaded areas indicate the standard error of the mean. Note that stronger HR responses followed the presentation of the fast-AM FM. (**E**) Area under the average HR curve calculated in 12 awake bats in response to the two treatments of the FM syllable. Note that the fast-AM treatment rendered the strongest HR responses. Area values have been z-scored for illustration purposes due to the HR variability across animals (z-scoring does not affect the results of paired statistics reported). (**F**,**G**) Show similar results as those reported in (**D**,**E**), but in response to the quasiconstant-frequency/frequency-modulated (qCF-FM) syllable and its demodulated treatment. (**H**,**I**) Show the results obtained when the sinusoidally frequency modulated syllable (SFM) was used as stimulus.

The final stimuli presented to the bats were sequences of either natural fAMVs or artificial-sAMVs in which the same sound was repeated 66 times in the form of 11 bouts (Fig. 7E, top panel), with 6 repetitions of the same syllable per bout (see Fig. 7E, bottom panel). The inter-bout interval was fixed to 80 ms and, within bouts, syllables were repeated at intervals of 14 ms. These parameters were chosen based on median values reported in a quantitative study on the structure of *C. perspicillata’s* distress sequences^24^. HR changes in response to the acoustic signals described above were measured by attaching three electrodes (active, reference and ground) to the left and right sides of the chest and to the back of the animals, respectively (Fig. 8A). The resulting voltage differences were measured and the location of QRS complexes were detected automatically based on their amplitudes (Fig. 8B). Instantaneous HR was then calculated considering the interval between consecutive QRS complexes and expressed in beats/min. HR was measured from 5 s before (baseline) until 10 s after stimulus presentation.

### Listening to fast amplitude modulated vocalizations boosts the bats’ heart rate

As mentioned in the preceding text, acoustic stimulation is known to increase the bats’ HR^31,49^. This effect was also visible in our data, as illustrated in Fig. 8C (left) for two stimulation trials in which sequences of FM syllables were presented to an awake bat, with the syllables occurring either in their natural (with fast modulations) or demodulated forms (Fig. 8C left and right, respectively). Note that the example individual trials presented in Fig. 8C already point towards larger HR increments in response to rough-like than to demodulated sounds.

Average HR curves obtained by pooling data from all stimulation trials in all bats tested are shown in Fig. 8D,F,H (12 bats; 10 trials per animal and stimulus treatment, trials with movement artifacts were not considered, see methods). Regardless of the syllable analyzed, the natural treatment containing fast amplitude modulation always produced higher HR increments than the demodulated treatment of the corresponding syllable. This was statistically validated by comparing the area under the HR curve in the first 10 s after stimulation in each bat using paired statistics (Fig. 8E,G,I, n_bats_ = 12, FM syllable: p_signrank_ = 0.002, qCF-FM syllable: p_signrank_ = 0.00009, SFM syllable: p_signrank_ = 0.0004). Altogether the data obtained indicates that the presence of fast amplitude modulation (putative roughness) make signals more effective in accelerating the bats’ HR. The latter points towards a role of fast amplitude modulation for influencing the listeners’ physiology.

### Listening to fast amplitude modulated vocalizations triggers frequency-following responses in the bat brain

Our results show clear evidence on the existence of fast amplitude modulation ~1.7 kHz in bat distress vocalizations. Hearing rough-like sounds accelerates the bats’ heart rate. For the latter to occur, amplitude modulation patterns related to roughness-like acoustic regimes must be represented in the bats’ brain. We investigated whether frequency-following responses (FFRs) occurred in response to fAMVs. FFRs appear as rhythmic brain signals occurring at the same frequency of the sensory input (i.e. 1.7 kHz in fAMVs). In humans and other animal species, FFRs have been used to study the auditory system’s ability to process temporal periodicities^50–54^.

**Figure 9:**
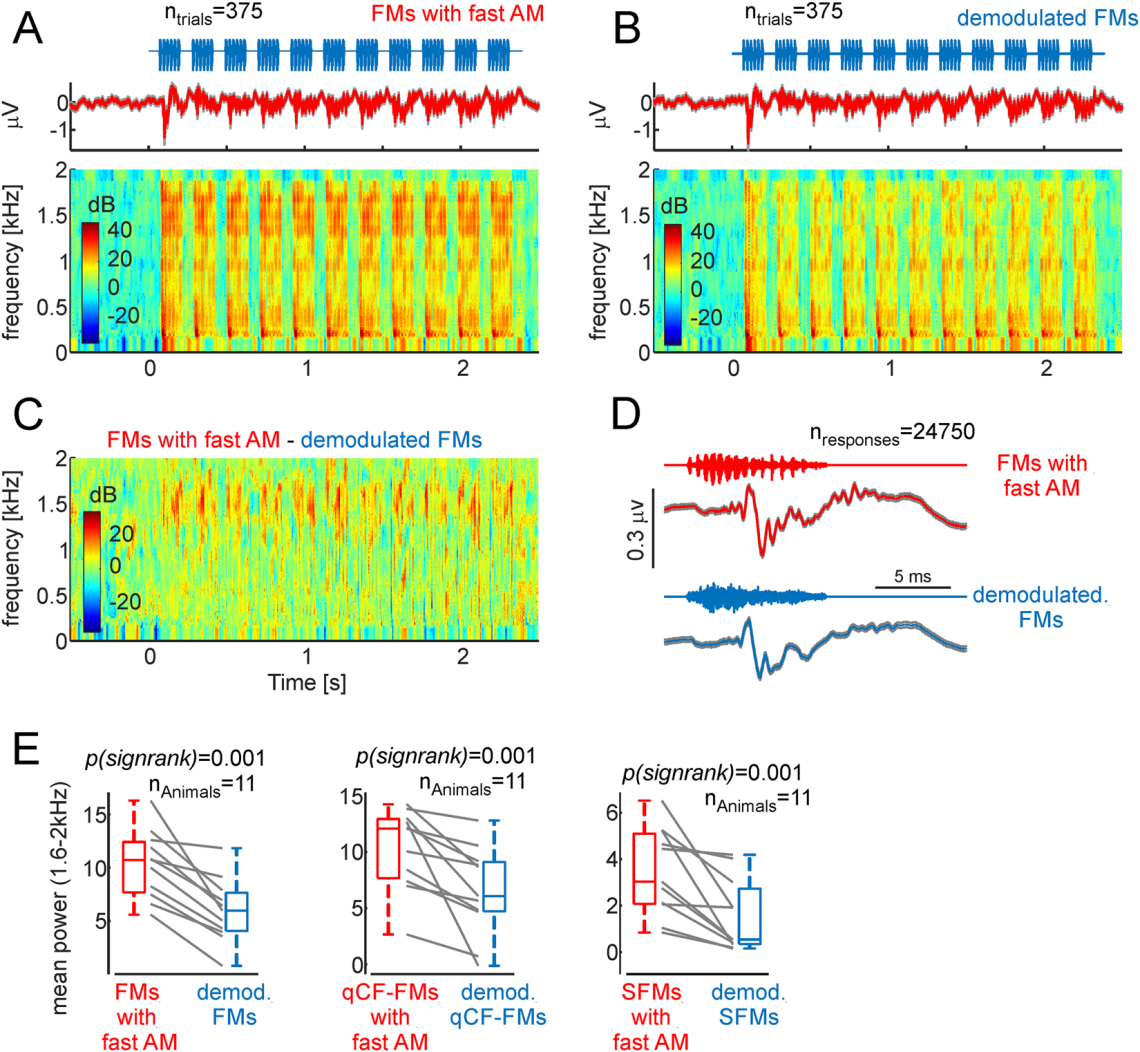
Fast amplitude modulated (AM) vocalizations trigger frequency following responses in the bat brain. (**A**) Average neural responses to a sequence of fast-AM FM syllables across all trials and animals (n = 11) studied. Responses are represented as voltage vs. time and in the form of neural spectrograms. (**B**) Same as panel A, but in response to the sequence of demodulated FMs. Note that at frequencies ~1.7 kHz, more power occurred in response to fast-AM than to demodulated FMs. (**C**) Difference between the neural spectrograms depicted in (**A**,**B**). (**D**) Voltage fluctuations obtained after averaging neural responses to each fast-AM FM (red) and each demodulated FM syllable (blue) across trials and animals. Note that responses to fast-AM FMs carried faster modulations than those obtained in response to demodulated FMs. (**E**) Mean power in the range from 1.6–2 kHz across animals and sequences studied. In each animal, fast AM syllables rendered higher power than their demodulated treatment.

FFRs were studied by measuring intracortical electroencephalogram signals (iEEG, active electrode placed over the auditory cortex) in 11 head-restrained, awake bats. As stimuli, the same sequences of natural fAMVs and demodulated syllables used for measuring HR (see Fig. 7) were presented. Figure 9A,B show the average iEEG obtained across animals in response to the sequence of FMs carrying amplitude modulation at 1.7 kHz (Fig. 9A, top panel) and demodulated FMs (Fig. 9B, top panel). Spectrograms of the signals recorded were calculated to assess the power relations at the FOIs in the neural responses to rough-like and demodulated FMs (Fig. 9A,B, bottom panels). From the spectrografic representations, it is clear that responses evoked by natural fAMVs had high power in frequencies close to 1.7 kHz. The latter becomes obvious after subtracting the two spectrograms (Fig. 9C). Such pronounced power at frequencies close to 1.7 kHz is likely related to the occurrence of an FFR that represents the bat auditory system’s ability to represent fast amplitude modulations occurring in distress sounds (see below for a discussion of possible neural sources). The FFR can also be visualized as fast fluctuations in the neural signals obtained after averaging the 20 ms time-window following the presentation of each syllable across sequences, trials and animals (Fig. 9D, n = 24950 responses to the FM syllable).

To statistically validate the presence of FFRs in response to rough-like syllables, the average power (across time) at the FOIs was calculated in the neural spectrograms obtained for each animal in response to each of the six sequences studied (sequences composed of: (1) natural modulated FM syllable, (2) demodulated FM syllable, (3) natural modulated qCF-FM syllable, (4) demodulated qCF-FM syllable, (5) natural modulated SFM syllable, (6) demodulated SFM syllable). Average neural spectrograms corresponding to responses to the qCF-FM and SFM syllables can be found in Supplementary Fig. S5. At the population level, when considering the power of the neural responses in the range from 1.6 to 2 kHz, there were significant differences between responses to natural fAMVs and demodulated vocalizations in all three cases studied (p_signrank_ = 0.001 for all three cases studied, Fig. 9E). The latter indicates that the bat auditory system is capable of representing the fast amplitude modulations found in rough-like sounds using a temporal code.

## Discussion

The main aim of this article was to study the temporal modulation pattern of distress syllables produced by bats, a highly vocal animal group. We tested the idea that fast amplitude fluctuations (roughness-like patterns) could be a generalized trait of mammalian vocalizations produced in distress contexts. If this hypothesis was true, then, a large percentage of bat distress vocalizations should carry fast temporal periodicities characteristic of the roughness regime.

Four main observations support our driving hypothesis. (i) Almost half (47.7%) of the distress syllables produced by bats (species *C. perspillata*) carry amplitude modulations at ~1.7 kHz and the percentage of rough-like vocalizations is much lower during social interactions (6.3%). (ii) 1.7 kHz (putative bat roughness) is not present as carrier frequency in bat vocalizations and, in addition, this frequency does not evoke responses in the bats’ cochlea based on non-linear mechanics. (iii) Vocalizations carrying fast amplitude modulation produce larger heart rate increments than their demodulated versions, thus suggesting that sounds carrying rough-like patterns can indeed elicit alarm state in bats. (iv) Rough-like vocalizations evoke frequency following responses in the brain, suggesting that the bats’ auditory system can represent fast amplitude modulations based on a temporal code.

### Comparison with previous studies

Several studies in bats and other animal species have characterized the amplitude modulation pattern of natural vocalizations. A recent study in the bat species *Phyllostomus discolor* (a sister species of *C. perspicillata*) described vocalizations carrying amplitude modulations at rates close to 130 Hz^55^. Periodicity values below 500 Hz also have been described in previous studies in frogs and birds^56–61^, although some of these periodicities have been linked to acoustic correlates of pitch rather than to amplitude modulation. In humans, amplitude fluctuations occur in screamed vocalizations at amplitude modulation rates between 30–150 Hz^1^. The periodicity values reported in the present article reach 1.7 kHz, that is, >10 times faster than modulation rates reported in human screams and at least 8 times faster than modulation rates reported in other vertebrates^57,60^, including other bat species^55^. Note that describing a sound as rough implies a perceptual evaluation of the acoustic waves heard. Throughout this article we referred to rough-like or fast amplitude modulated sounds because we cannot know what other animals perceive when they listen to amplitude modulation. It is possible that amplitude modulation frequencies linked to the perception of roughness differs across species. If this is the case, then researchers need to re-define the concept of “roughness” to fast amplitude modulations or transients that (at least in humans) remain perceived as discrete events (i.e. below the pitch percept^62^).

Our data suggests that amplitude modulations at ~1.7 kHz could be a hallmark feature of distress calling in bats, since the percentage of rough-like vocalizations is much lower in social contexts (distress: 47.7% vs social: 6.3%). Note that our definition of social context is quite broad and could include several types of interactions between bats such as grooming, mating, aggression, appeasement, among others. Our assumption was that non-aggressive interactions would prevail in the group of bats studied. The latter was ensured by waiting 7 days before conducting acoustic recordings. Though likely fulfilled, our assumption is impossible to prove without video feeds depicting bat interactions. Future studies could try to quantify the occurrence of amplitude modulations in call types matched to specific behaviors. Such approach has been used before but without looking specifically at the pattern of amplitude modulation of single syllables^35,63–66^. In humans, acoustic roughness is not specific to fearful screams. This feature also has been found in other harsh sounding vocalization types such as infant cries^3^. The same could be true for bats.

Bats and humans are phylogenetically distant species that do not share common ecological niches. Yet, in both species fast temporal periodicities are present in vocalizations emitted in distressful contexts. According to our data, in bats, the average ratio between peak frequency (the main carrier frequency) and amplitude modulation frequency is equal to 15.2 (see Results). It is difficult to estimate this ratio for human screams. The original study describing human roughness (ref. ^1^) did not report peak frequency values of screamed vocalizations. A recent study on human screams did report peak frequency and roughness values of screams (ref. ^2^) but they estimated roughness using a different approach that renders unitless values. Considering 21 sounds that were classified as screams by at least 95% of the participants in the Schwartz *et al*. study^2^, one can calculate an average mean peak frequency of 1929 Hz. If all these sounds carried roughness at values ~90 Hz (the center of the roughness regime reported by Arnal *et al*.^1^, we could calculate a peak frequency/roughness ratio of 19.3 for human participants. The ratio calculated from bat data (15.2, this study) and that calculated from human data (after estimations) do not appear to be far off from each other. A possible interpretation of this result is that the two species separate carrier and modulator frequencies by a similar ratio. Future studies could explore what specializations (if any) exist in the auditory system for processing carriers and modulators separated by this ratio.

We want to point out that although a roughness-like acoustic regime is a likely candidate for explaining the fast amplitude modulations observed in the bat vocalizations, temporally periodic structures leading to complex spectra could also be related to non-linear phonation phenomena such as “torus” and “deterministic chaos”^67–69^. In bats, these two explanations (roughness-like patterns and non-linear phenomena) might not be mutually exclusive. Non-linear phenomena are identified based on the sounds’ spectrograms while acoustic roughness is identified based on the sounds’ oscillogram. Therefore, it is difficult to disentangle between these two phenomena (roughness vs. vocal non-linearities). In other words, it remains open if roughness and non-linear phenomena are the same thing measured in different ways.

Previous studies in bats have reported the occurrence of non-linear phenomena^64,66^. Particularly deterministic chaos appears to be linked specifically to high aggression contexts^66^. The occurrence of non-linearities during vocalization production is ubiquitous in vertebrates^64,66,69–72^. It has been argued that non-linear sounds result from saturation in the vocal production apparatus, and that their generation does not require complex neural control mechanisms^67,69^. Yet, non-linearities occur in sounds uttered by several vertebrate species, including human infants, and they capture the listeners’ attention due to their non-predictable structure (for review see ref. ^67^). It has also been argued that non-linear sounds prevent behavioral habituation^73^. The presence of spectro-temporally complex sounds (like the rough-like vocalizations reported here) within bat distress broadcasts could make emitted signals more successful in grabbing the listeners’ attention. Most bat distress sequences are long (>1 s) and repetitive, since the same syllable spectro-temporal design is used throughout the broadcast^24,65^. Our data indicates that rough-like syllables occur at different positions within distress sequences but there is a preference for these sounds to occur at the beginning of the broadcasts (see Fig. 2G). One could speculate that a listener exposed to sequences with transitions between rough and non-rough sounds could experience less neuronal adaptation of responses to the individual syllables. Future studies could explore this possibility by studying phenomena such as stimulus specific adaptation^74,75^ in response to combinations of fast and slow temporally modulated sounds at the neuronal level.

Note that the present study presents evidence on the occurrence of rough-like patterns during distress calling in bats and the effect of these calls on the listeners’ physiology and brain processes, but we do not present any evidence of bats producing these sounds “intentionally”. It has been suggested that in humans, growl-like voices (related to anger) could be a byproduct of abdominal muscle contraction which changes resonances in the vocal tract^4^. Abdominal muscle contraction is a mechanism for enhancing spine stability^76^, which in turn is fundamental for achieving advantageous postures to produce and/or withstand physical attacks in distressful contexts. The mechanism that accounts for growl-like voices in humans is largely reactive and could account as well for roughness in human screams and even for the fast amplitude modulations reported in the present study in bat distress calls.

### Possible neural mechanisms for roughness extraction

Our data shows that the presence of roughness accelerates the heart rate of awake bats. This indicates that fast amplitude fluctuations are extracted somehow in the bats’ brain. Note that the audiogram of most bat species is shifted towards ultrasonic frequencies (>20 kHz). For example, auditory thresholds in *C. perspicillata* have values above 70 dB SPL for frequencies below 10 kHz^39,41^. In fact, the DPOAE measurements presented here showed no cochlear responses to low frequency sounds below 5 kHz. According to our data, sound pressure level measured at ~1.7 kHz is very low (average values ~0 dB SPL), which further hampers its representation at the cochlear level.

Two possibilities come to mind when thinking about neural strategies for coding fast periodicities related to roughness: (i) the use of spectral harmonic codes and (ii) temporal codes^77,78^. Spectral harmonic coding does not depend on a region of the cochlea being able to extract low frequencies that are poorly represented as carriers but rather on the ability of the cochlea to resolve closely placed harmonics of the modulator^57^. Whether the cochlea of *C. perspicillata* can resolve harmonics separated by 1.7 kHz remains to be tested. It has been argued that the exact periodicity value at which a switch from temporal to spectral coding occurs might differ across species^57^. At least in the auditory nerve of squirrel monkeys, spiking activity can statistically lock to the occurrence of periodicity cycles for frequencies up to 5 kHz (temporal coding^79^). If the same is assumed for bats, then the 1.7 kHz modulation shown here could be encoded in auditory nerve activity patterns. FFR measurements reported in this manuscript are in agreement with this idea.

We show that surface potentials represent the fast temporal periodicities occurring at frequencies ~1.7 kHz based on a temporal code. Note that FFRs in response to amplitude fluctuations faster than 1 kHz are not unique to bats^51^. Our recordings were based on surface potentials (iEEG) that are suited for studying whole-brain activity, but are not ideal for identifying possible generators contributing to the neural signal measured. iEEGs can be influenced even by signals such as the cochlear microphonic, reflecting the response of hair cells rather than central neural generators^80^. Previous studies measuring FFRs in humans concluded that FFRs obtained in response to fast frequencies (i.e. >100 Hz) typically result from activity in subcortical structures ^53,81,82^. The same could be true for *C. perspicillata*, since in this species most auditory cortex neurons cannot track amplitude modulations above 20 Hz^30,83^, even though field potentials measured at the cortical level do entrain to faster acoustic rhythms^27,28^. Note that structures outside the classical ascending auditory pathway could also be involved in the representation of rough sounds. For example, the amygdala is a likely candidate for providing such representations. In humans, this structure is differentially activated by screamed and non-screamed sounds^1^. In bats, electric stimulation of the amygdala triggers changes in heart rate^48^. It is thus plausible to suggest an involvement of the amygdala in the elevated HRs reported in this article in response to rough sounds.

Taken together, the findings reported in this manuscript indicate that bats can utter sounds that carry superfast temporal modulations in the order of kHz. Such sounds are more likely to occur in distress contexts and, albeit many differences, they share similarities with the acoustic correlates of roughness found in human screams^1,2^. Rough-like sounds are represented in the bats’ auditory system by means of frequency following responses and they accelerate the bats’ heart rate, an autonomic response to alarm signals that could be instrumental for the bats’ survival.

## Methods

### Distress call recording and analysis

All the experiments described in this article were carried out in accordance with current laws for animal experimentation in Germany (permit approved by the Regierungspräsidium Darmstadt, Germany, permit # F104/57) and with the declaration of Helsinki. Distress vocalizations were recorded from 13 adult bats (6 females and 7 males) of the species *C. perspicillata*. Bats were captured in a breeding colony at the Institute for Cell Biology and Neuroscience (Frankfurt University) and brought one by one into an acoustically isolated chamber where the distress vocalization recordings took place. Methods used in this article for recording distress calls have been described elsewhere^24^. In a previous article, we focused in studying the properties of distress “sequences” without considering the presence of rough-like patterns within individual syllables. The latter is the main focus of this paper.

For acoustic recordings, animals were hand-held with their face pointing straight into a microphone (Brüel&Kjaer, ¼-inch Microphone 4135, Microphone Preamplifier 2670) located at 1.5 m from the bat. To encourage the production of distress calls, the researcher holding the animal softly caressed the neck-skin of the bats. Recordings lasted up to 3 min per bat. The recording microphone was powered via a custom-built microphone amplifier and connected to a commercially available sound acquisition system (UltraSoundGate 116Hm mobile recording interface, +Recorder Software, Avisoft Bioacoustics, Germany) for sound digitization at 300 kHz (16-bit precision). Digitized signals were stored in a computer for offline analysis using the Avisoft SAS Lab Pro software (v.5.2 Avisoft Bioacoustics, Germany). The temporal position of individual “syllables” in each recording was automatically detected using an amplitude threshold of 4.1% of the maximum recording amplitude allowed when recording with the microphone amplifier gain set to the minimum. A syllable was defined as a fluctuation in amplitude in which the signal level did not drop below the amplitude threshold criterion (the 4.1% mentioned above) for a period of at least 1 ms. Amplitude detection was manually revised for each syllable to ensure the accuracy of the results.

The temporal modulation spectrum (TMS), frequency spectrum, spectrogram, and modulation power spectrum (MPS) of each syllable were calculated and used for acoustic analysis. TMS was calculated as the FFT of each syllable’s amplitude envelope (secant method, temporal resolution = 0.1 ms). Frequency spectra were calculated as the FFT of each syllable’s waveform and interpolated to a resolution of 200 Hz for averaging purposes, using a linear interpolant. Short time Fourier transforms (STFTs) were calculated on zero-padded signals (0.5 s padding) using the following parameters: window length = 64, number of FFT points = 64, hop =1. The sampling rate was equal to 300 kHz. Zero-padding was necessary for obtaining STFTs of similar temporal and spectral resolutions across the syllables studied. The STFTs obtained were then used for computing modulation power spectra (see below).

For syllable classification based on their TMS, a binary support vector machine (SVM) classifier was used. The SVM classifier was trained (*fitcsvm* function, rbf kernel, Matlab 2018, no standardization) using the TMS of 100 vocalizations: 50 vocalizations contained pronounced periodicities in the range from 1.1–2.5 kHz, and another 50 vocalizations had no pronounced power in their TMS for that frequency range (see training TMS sets in Supplementary Fig. S1). The vocalizations chosen for the training sets were randomly picked after visual inspection of the entire dataset. The model cross-validation error (calculated using 10-fold cross-validation) amounted to 2%.

Harmonic to noise differences (HND) were used to complement classic spectral analysis. HNDs were calculated as the difference between the observed- and smooth-FFT of each syllable. The smooth-FFT was obtained using a 5-point moving average filter that removed peaks in the observed-FFTs. All FFTs had a frequency resolution of 200 Hz. The latter was achieved by linear interpolation of the FFTs obtained from each sound. This was a necessary step, since frequency resolution is linked to sound length. The HND of each sound was equal to the maximum absolute difference between the observed and smoothed FFTs. To characterize the presence of spectral regularities spectral autocorrelograms were used. To that end, the spectrum of each syllable was autocorrelated for frequencies of up to ±10 kHz. The median interpeak distance (MIPD) was used to measure regularity values. MIPDs were obtained after detecting local peaks in the autocorrelograms’ local maxima using the peakseek function (peak prominence = 0.025). This procedure was effective (i.e. it detected more than one peak) in 2699 out of 3349 fAMVs detected (80.6%). In the remaining fAMVs, the spectra were too noisy for local peak detection.

### Computing modulation power spectra

The MPS represents each syllable in the spectral and temporal modulation domains (see Fig. 6) and it was calculated as the 2D-FFT of the log-transformed STFTs. The absolute value of the 2D-FFT was then squared and log-transformed to produce the MPS. Note that spectrogram parameters (i.e. number of FFT points, window length and hop, see above) were chosen so that the temporal resolution of spectrographic representations was precise enough for representing amplitude modulation values around 1.7 kHz in the temporal modulation domain. Using a larger window size could have resulted in periodicity representations in the spectral modulation domain, rather than in the temporal domain. STFTs were calculated using a linear frequency axis thus rendering spectral modulations in the MPS expressed in cycles/kHz. STFTs obtained with a log frequency axis result in spectral modulations given in cycles/octave. Previous studies have suggested that MPS representations in cycles/kHz are useful when dealing with harmonic sounds, as it was the case here^47^.

For constructing the stimuli used in ECG and iEEG experiments, three natural rough syllables (see Figs. 7 and S4) were demodulated using an MPS filtering algorithm similar to that described in previous studies in humans^47^. Before MPS filtering the three syllables used as stimuli were downsampled to 192 kHz. MPS filtering was achieved by nullifying all MPS-power across spectral modulations in the temporal modulation range from −1 to −4 kHz and from 1 to 4 kHz. This temporal modulation range covered the fast periodicities of interest, occurring at frequencies at ~1.7 kHz. The filtered MPS was then exponentiated, root-mean squared, and transformed into a matrix of complex numbers, built taking into account the phase matrix obtained from the 2D-FFT of the original sounds. The resulting matrix was then transformed into an STFT using an inverse FFT2 procedure. The resulting STFT was then exponentiated and transformed into a sound waveform using an inverse STFT, implemented based on an inverse FFT and the weighted-overlap-add method^84^. The new demodulated sound and the natural fAMV from which it derived were then root-mean-square normalized to avoid level differences. The sound synthesis procedures described above involve inverse STFTs that could be affected by time-frequency trade-offs. To quantify possible errors during sound synthesis using inverse STFTs we used a method proposed in previous studies^47^, in which the difference between the desired and observed STFTs are squared and divided by the desired STFT. The observed STFT is obtained as the STFT of the newly synthetized sound, while the desired STFT is obtained after exponentiation of the outcome of the inverse STFT obtained from the filtered MPS. For all three sounds used, the synthesis error was below 2%.

### Setup for ECG measurements

The natural fAMVs and their demodulated versions obtained from MPS filtering were used to build acoustic stimulation sequences as described in Fig. 7. For sequence building, the start and end of each syllable was multiplied by a linear fading window of 0.2 ms to avoid acoustic artifacts during stimulation. Sounds were synthetized in MATLAB 2015 (The MathWorks, Inc., Natick, Massachusetts, United States), produced through a sound card (RME Fireface 400, sampling rate = 192 kHz), amplified (Rotel power amplifier, RB-850) and played from a speaker (NeoCD 1.0 Ribbon Tweeter; Fuontek Electronics, China) placed 15 cm in front of the bats’ nose. The RMS level of the 6 syllables (3 natural fAMVs and 3 demodulated syllables) when produced by the speaker spanned between 68.6 and 70.5 dB SPL (mean = 69.3 dB SPL, std = 0.7 dB SPL). To prevent adaptation, sequences were played randomly at intervals of 3 min between each sequence presentation. All measurements were conducted inside a soundproofed chamber.

Electrocardiogram (ECG) measurements were conducted in 12 awake animals (5 females, 7 males) placed on a custom-built holder similar to those used in electrophysiology experiments^27,85,86^. ECG signals were obtained by placing three electrodes (active, reference, and ground) on the bats’ chest and back (see Fig. 8A). We found this configuration to be more stable for ECG recordings in awake bats than configurations involving the thumbs and legs^87^. The three electrodes were attached to the inside of a custom-built Velcro belt. Electrolytic gel (Supervisc, EasyCap GMBH, Germany) was used to improve the contact between electrodes and skin. After the experiments, the skin was carefully cleaned using cotton-swabs and water.

Measuring electrodes were attached to a pre-amplifier/amplifier system (EX1 Differential amplifier, Dagan Corporation). Signals were amplified (gain = 50) and band-pass filtered by the recording amplifier between 0.1 kHz and 1 kHz. ECG signals were digitized using the same sound card used for acoustic stimulation (see above), down-sampled to 9.6 kHz, and stored in a computer for offline analysis. To facilitate the automatic detection of QRS complexes (see below) the signal was adjusted so that the largest amplitude deflection recorded had a negative sign. The instantaneous heart rate was calculated as the inverse of the interval between consecutive QRS complexes multiplied by 60, to express it in beats/min. QRS complexes were identified by setting an amplitude threshold that detected the QRS events as “spikes” whose amplitude was larger than at least two standard deviations of the noise level calculated from the envelope of the ECG signal.

Altogether, we presented 10 trials of each syllable and stimulus treatment (natural and demodulated) amounting to six different conditions. The 10 trials were split into two blocks with a break of 10 min between blocks during which water was offered to the animals. Awake bats occasionally moved during the recordings. Trials that contained movement artifacts were excluded from the analysis. In ECG recordings, movement artifacts appear as signal peaks (spikes) occurring at intervals shorter than 62 ms thus producing instantaneous frequencies above 960 beats/min. This value had been used in a previous article for movement detection^87^ and was used here for trial rejection. Overall, trials contaminated with movement artifacts represented 17% for the total number of trials gathered across all animals and stimuli tested (599/720). To average HR measurements across trials, instantaneous HR values were linearly interpolated with a temporal resolution 0.5 s.

### Setup for DPOAE measurements

DPOAEs were recorded in 6 adult awake *C. perspicillata* (3 males, 3 females) in a soundproofed chamber. To ensure that bats were not able to move during the recordings, their heads were fixed by holding a metal rod attached to the scalp. The surgical procedure for metal rod fixation has been described elsewhere^30,85,88^. Briefly, in fully anesthetized bats (Ketamine (10 mg *kg^−1^ Ketavet, Pfizer) and Xylazine (38 mg *kg^−1^ Rompun, Bayer)), the skin and muscles covering the scalp were removed. The scalp surface was cleaned, and a custom-made metal rod (1 cm length, 0.1 cm diameter) was then glued to the skull using dental cement (Paladur, Heraeus Kulzer GmbH).

The DPOAE setup followed the specifications described in previous studies^42,44^. To measure DPOAEs, an acoustic coupler was placed in the outer ear canal at a distance of about 0.3–1.0 mm from the tympanum under visual control (Zeiss OPMI 1-FR binocular, Carl Zeiss AG, Jena, Germany). The coupler consisted of three acoustic channels that converged at the coupler’s tip. Two of the coupler channels were connected to reversely driven condenser microphones used as loudspeakers (1/2″, MTG MK202, Microtech Gefell GmbH, Gefell, Germany) and the third channel contained a sensitive microphone (1/4″, B&K 4939, Brüel & Kjær, Nærum, Denmark) for recording DPOAEs. A soundcard was used to generate the two pure tone stimuli and to record DPOAEs (RME fireface UC, RME Audio AG, Haimhausen, Germany; sampling rate: 192 kHz). Data acquisition and data analysis programs were written in MATLAB (MATLAB 2015b, MathWorks Inc.). The sound system was calibrated *in situ* before each measurement using white noise. DPOAEs were recorded by varying the stimulus frequency f2 between 1 and 25 kHz (1 kHz steps) and between 1 and 3.2 kHz (0.2 kHz steps) to obtain DPOAE data at coarse and fine frequency resolution, respectively. The ratio between f2 and f1 frequencies was kept constant at 1.25. Two f2 levels were tested: 50 and 70 dB SPL (f1 level = 60 and 80 dB SPL, respectively). To calculate DPOAE amplitudes, FFT-analysis was performed from 100 averages of the time signal acquired based on 8192-point epochs. The noise floor was calculated as the arithmetic mean of the amplitude of 20 points in the spectrum taken on either side of the DPOAE frequency within a 100 Hz frequency span. This method yielded DPgrams (plots of DPOAE amplitude versus f2 frequency) that were used to confirm that hearing deteriorates in *C. perspicillata* for frequencies below 5 kHz.

### Setup for neurophysiology measurements

Intracranial electroencephalogram (iEEG) signals were measured to assess the occurrence of frequency following responses that could represent the fast periodicities found in fAMVs. iEEGs were measured in fully awake, head-restrained animals. The head of the bats was immobilized by holding a metal rod attached to the scalp. The surgical procedures used to attach the metal rod were similar to those described in the preceding text (see methods for DPOAE measurements). iEEGs were obtained using silver wires placed below the scalp. Three wires were used (active reference and ground). In each animal, the active electrode was placed over the primary auditory cortex, the reference was placed on a similar rostro-ventral position as the active electrode but close to the midline and the ground electrode was placed over the cerebellum. The location of the primary auditory cortex was estimated using external landmarks such as the medial cerebral artery and the pseudo-central sulcus^85,86,89^.

Measuring electrodes were attached to a pre-amplifier/amplifier system (EX1 Differential amplifier, Dagan Corporation). Signals were amplified (gain = 50) and band-pass filtered by the recording amplifier between 0.1 kHz and 5 kHz. iEEG signals were digitized using the same sound card used for acoustic stimulation (see above), downsampled to 9.6 kHz, and stored in a computer for offline analysis. The multi-taper method was used to estimate spectral power in the iEEG signals recorded^90^ (5 tapers, time-bandwidth product of 3). Neural signals are known to follow a power rule by which high frequencies contain less power than lower frequencies. For better visualization of the power at high frequencies (i.e. 1.7 kHz), neural spectrograms were corrected by subtracting the average power at each frequency during time periods of 1.5 s, in which no acoustic stimulation was presented.

### Statistical analysis

Statistical analysis was done in Matlab (Statistics toolbox, MATLAB R2015b, The MathWorks Inc., Natick, MA, 2015). Normality of data distributions was tested using the Kolmogorov-Smirnov test. Paired and un-paired non-parametric statistics (signrank and ranksum tests, respectively) were used throughout the manuscript since the data was not normally distributed. Size effects were calculated using the Cliff’s delta metric. Effect size groups were defined as proposed in previous studies^36^.

## Supplementary information

Supplementary information.
