# Supplementary material for: Bats distress vocalizations carry fast amplitude modulations that could represent an acoustic correlate of roughness
Source: Sci Rep. 2020 Apr 30;10:7332. doi: 10.1038/s41598-020-64323-7 (PMC7192923; doi:10.1038/s41598-020-64323-7)
Supplement: Supplementary file 1 — Supplementary information. [file 41598_2020_64323_MOESM1_ESM.pdf]

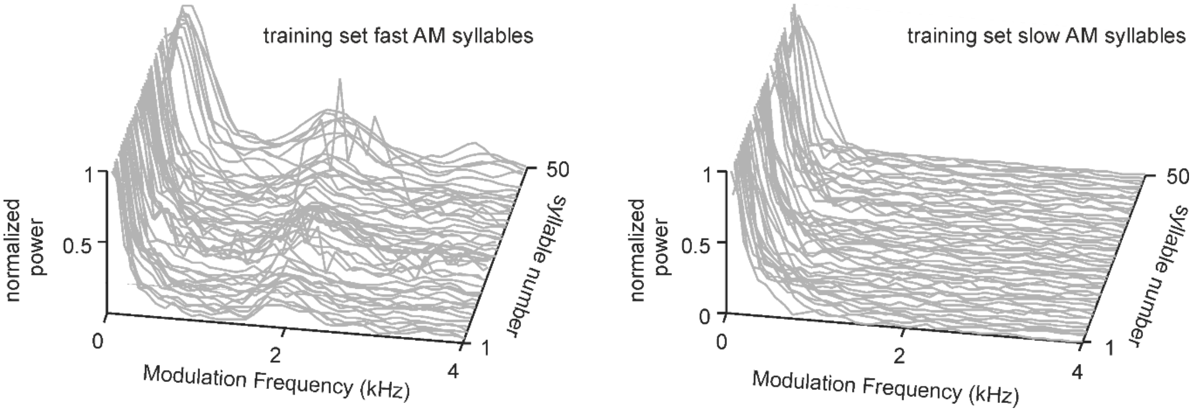

Figure S1. Temporal modulation spectra of vocalizations used to train the support vector machine classifier. The fast amplitude modulated (AM) set includes 50 randomly chosen vocalizations containing fast periodicities at ~ 1.5 - 2 kHz. The slow AM set includes 50 randomly chosen vocalizations that contained low power in the 1.5-2 kHz range.

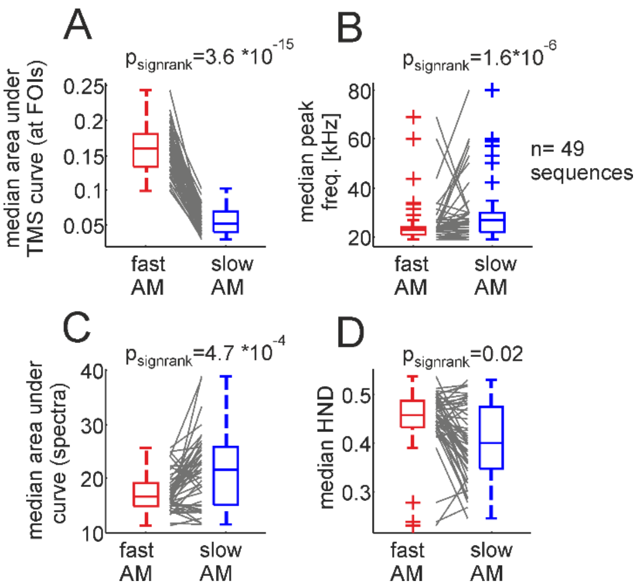

Figure S2. Comparing basic properties of fast and slow amplitude modulated vocalizations occurring within the same distress sequence, using paired statistics. Only data from sequences containing at least 10 fast-AM and 10 slow-AM calls were considered (N=49 sequences). The median of each acoustic parameter measured was calculated for all fast- and slow-AM calls occurring within the same sequence. The results of paired *signrank* tests are provided in each panel. A) Median area under the temporal modulation spectrum (TMS) at the frequencies of interest (FOIs = 1.15 to 2.45 kHz, the putative acoustic correlate of roughness in bats). B) The median peak frequency of fast- and slow-AM distress syllables. C) Median area under the spectra (a measure of frequency bandwidth). D) Median harmonic to noise difference. Note that statistical differences occurred in all cases, but they were strongest for the syllables' temporal modulation spectrum.

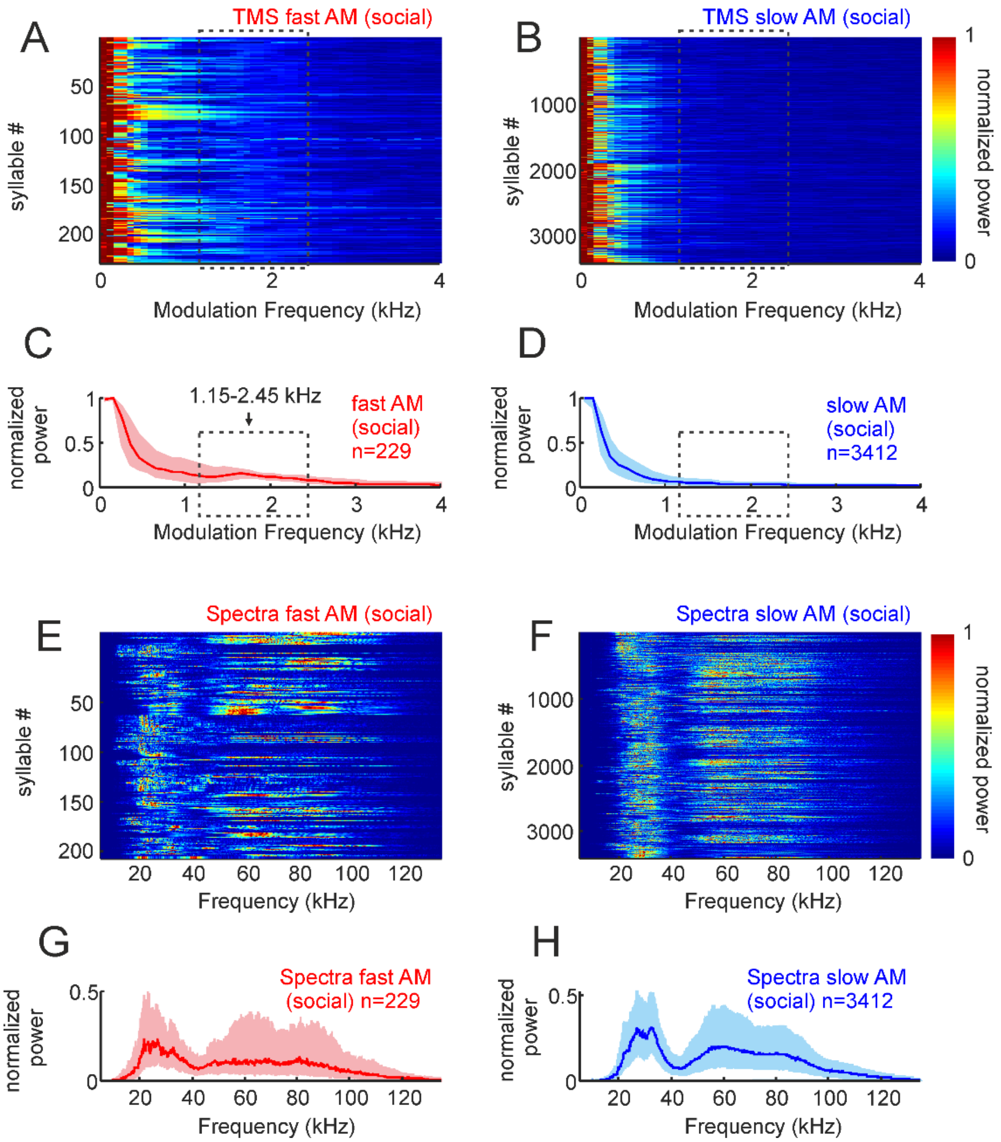

Figure S3. Temporal and spectral properties of fast and slow amplitude modulated (AM) vocalizations obtained while the animals were interacting in a keeping cage (social context). Syllables were split into fast and slow AM using the same support vector machine classifier used for classifying vocalizations emitted in the distress contexts (see also figure 2 in the results section). A and B show the temporal modulation spectrum (TMS) of the two syllable groups, represented as colormaps. C and D are median TMS of all fast and slow AM calls studied (25th and 75th percentiles shown as shaded areas). E and F show colormaps of the calls' spectra. G and H show the median spectra of the two syllable groups. Note that the percentage of fast AM calls emitted in the social context is very low (229 calls out of 3641 vocalizations studied, 6.3%).

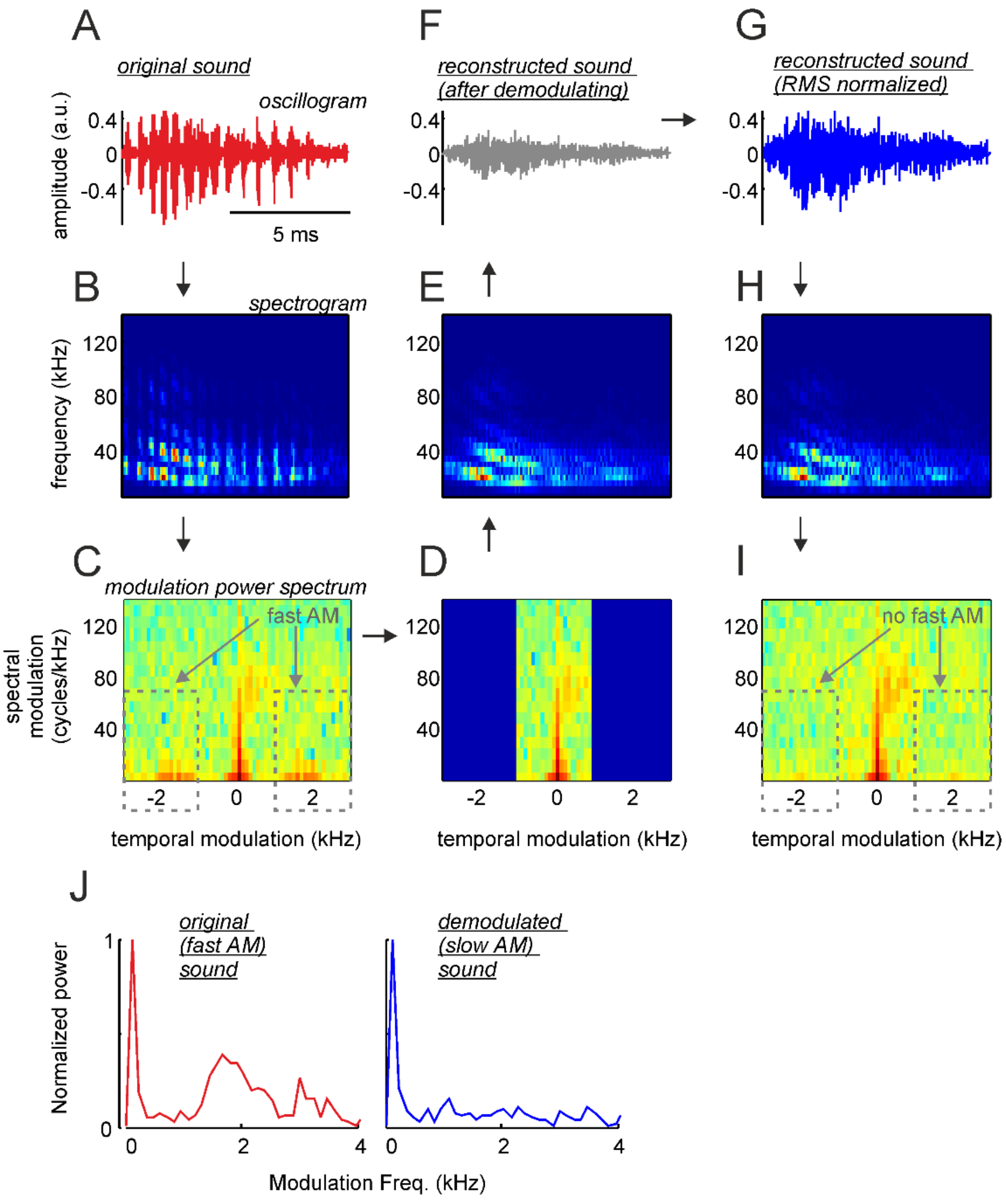

Figure S4. Pipeline used for demodulating fast amplitude modulated (AM) vocalizations. The waveform(**A**), spectrogram (**B**), and modulation power spectrum (MPS, **C**) of an example fast-AM syllable are shown. **D**: In the MPS, the power at modulation frequencies above 1 kHz and below -1 kHz was set to zero (blue areas in D). **E**: The short time Fourier transform (STFT) of the demodulated vocalization was constructed from the filtered MPS using an inverse FFT2 procedure. **F**: The waveform of the demodulated vocalization was obtained from the STFT represented in E using the weighted overlap add method (WOLA). **G**: The demodulated sound obtained was root-mean-square (RMS) normalized to match the energy of the natural fast-AM sound represented in A. **H**: Because the WOLA method can be affected by spectro-temporal trade-offs, the STFT of the demodulated sound shown in G was computed and compared to the "desired" STFT shown in E (see methods). The error was calculated as the squared difference between the desired STFT (shown in E) and the observed STFT (shown in H) divided by the values in the desired STFT. The resulting error value in this case was 1%. **I**: the MPS of the

demodulated sound. **J**: The temporal modulation spectra of the natural FPV (left) and its demodulated treatment (right).

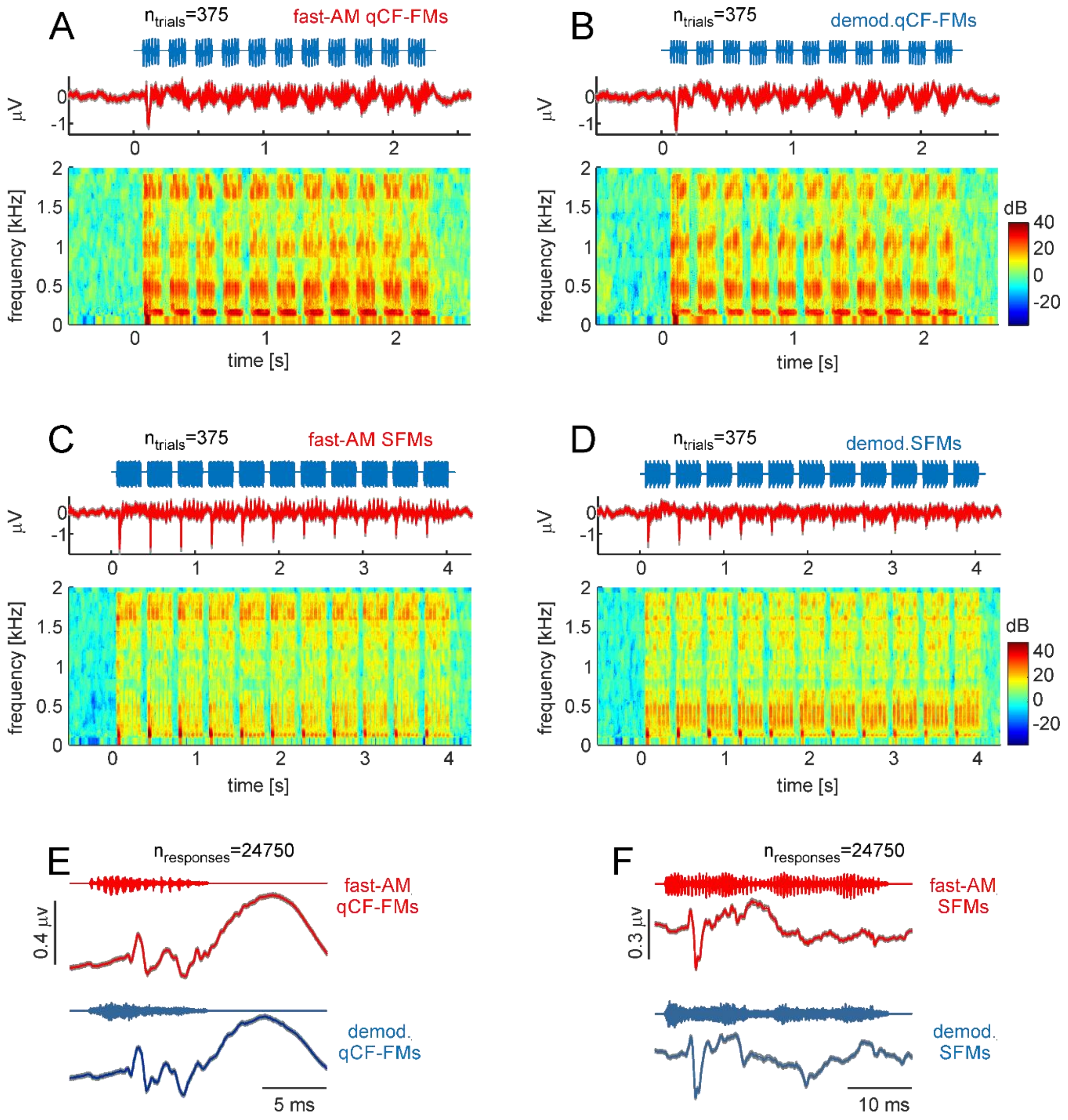

Figure S5. Neural responses to fast amplitude modulated (AM) and demodulated syllables. (**A** and **B**) Neural responses to a sequence of fast-AM qCF-FM syllables (A) and demodulated qCF-FM (B). Responses are represented as voltage vs. time and in the form of neural spectrograms. (**C** and **D**) Same as A-B but in response to sequences of fast-AM and demodulated SFMs. (**E**) Voltage fluctuations obtained after averaging neural responses to each fast-AM qCF-FM (red) and each demodulated qCF-FM syllable (blue) across trials and animals. (**F**) Same as E but in response to SFM syllables.
